# Molecular Evolution and Organization of Ribosomal DNA in the Hawkweed Tribe Hieraciinae (Cichorieae, Asteraceae)

**DOI:** 10.3389/fpls.2021.647375

**Published:** 2021-03-12

**Authors:** Judith Fehrer, Renáta Slavíková, Ladislava Paštová, Jiřina Josefiová, Patrik Mráz, Jindřich Chrtek, Yann J. K. Bertrand

**Affiliations:** ^1^Institute of Botany, Czech Academy of Sciences, Průhonice, Czechia; ^2^Department of Botany, Charles University, Prague, Czechia

**Keywords:** 5S rDNA, 45S rDNA, *Andryala*, concerted evolution, *Hieracium*, *in situ* hybridization, molecular phylogeny, *Pilosella*

## Abstract

Molecular evolution of ribosomal DNA can be highly dynamic. Hundreds to thousands of copies in the genome are subject to concerted evolution, which homogenizes sequence variants to different degrees. If well homogenized, sequences are suitable for phylogeny reconstruction; if not, sequence polymorphism has to be handled appropriately. Here we investigate non-coding rDNA sequences (ITS/ETS, 5S-NTS) along with the chromosomal organization of their respective loci (45S and 5S rDNA) in diploids of the Hieraciinae. The subtribe consists of genera *Hieracium*, *Pilosella*, *Andryala*, and *Hispidella* and has a complex evolutionary history characterized by ancient intergeneric hybridization, allele sharing among species, and incomplete lineage sorting. Direct or cloned Sanger sequences and phased alleles derived from Illumina genome sequencing were subjected to phylogenetic analyses. Patterns of homogenization and tree topologies based on the three regions were compared. In contrast to most other plant groups, 5S-NTS sequences were generally better homogenized than ITS and ETS sequences. A novel case of ancient intergeneric hybridization between *Hispidella* and *Hieracium* was inferred, and some further incongruences between the trees were found, suggesting independent evolution of these regions. In some species, homogenization of ITS/ETS and 5S-NTS sequences proceeded in different directions although the 5S rDNA locus always occurred on the same chromosome with one 45S rDNA locus. The ancestral rDNA organization in the Hieraciinae comprised 4 loci of 45S rDNA in terminal positions and 2 loci of 5S rDNA in interstitial positions per diploid genome. In *Hieracium*, some deviations from this general pattern were found (3, 6, or 7 loci of 45S rDNA; three loci of 5S rDNA). Some of these deviations concerned intraspecific variation, and most of them occurred at the tips of the tree or independently in different lineages. This indicates that the organization of rDNA loci is more dynamic than the evolution of sequences contained in them and that locus number is therefore largely unsuitable to inform about species relationships in *Hieracium*. No consistent differences in the degree of sequence homogenization and the number of 45S rDNA loci were found, suggesting interlocus concerted evolution.

## Introduction

The Cichorieae subtribe Hieraciinae is well defined on molecular and morphological grounds (Fehrer et al., 2007a; Krak and Mráz, 2008). Genera of the subtribe are *Hieracium* s.str., *Pilosella* (formerly treated as a subgenus of *Hieracium*, Bräutigam and Greuter, 2007), *Andryala* and monotypic *Hispidella* (Kilian et al., 2009). The basic chromosome number of all Hieraciinae is *x* = 9, with diploid representatives having *2n* = *2x* = 18. The mainly European genera *Pilosella* and *Hieracium* comprise many polyploid taxa, most or all of which reproduce apomictically, i.e., they form seeds without fertilization resulting in progeny corresponding to the maternal genotype (Krahulcová et al., 2000; Mráz and Zdvořák, 2019). Distribution ranges of diploids of both genera are usually small and often constrained to glacial refugia (Merxmüller, 1975). *Andryala* is an entirely diploid genus with its main distribution in Macaronesia and the Mediterranean region (Ferreira et al., 2015). *Hispidella hispanica* is also diploid and occurs in the central and western parts of the Iberian Peninsula (Tutin et al., 1976).

Phylogenetic relationships within the Hieraciinae have been previously inferred based on the internal transcribed spacer (ITS) region and the external transcribed spacer (ETS) of nuclear ribosomal DNA (rDNA) as well as on several chloroplast and low-copy nuclear markers (Fehrer et al., 2007a, 2009; Krak et al., 2013; Ferreira et al., 2015; Chrtek et al., 2020). ITS and ETS (the 5′ part of the intergenic spacer) are non-coding parts of the tandemly repeated 18S-5.8S-26S rDNA cistron, whose organization is the same in most organisms (Rogers and Bendich, 1987; Hillis and Dixon, 1991), namely ETS-18S-ITS1-5.8S-ITS2-26S. It is also referred to as 45S rDNA (sometimes 35S or 25S), which is commonly used as a cytogenetic marker (Denduangboripant et al., 2007; Garcia et al., 2010; Lan and Albert, 2011). The tandemly repeated 5S rDNA gene usually occurs separately from the 45S rDNA array in other regions of the genome in plants and animals (Hemleben and Grierson, 1978; Long and Dawid, 1980; Appels and Honeycutt, 1986; Wicke et al., 2011; but see Garcia et al., 2007, 2017 for exceptions), and the non-transcribed spacer (NTS) separates its individual units. The NTS is highly variable in plants (Cronn et al., 1996; Kaplan et al., 2013; Mahelka et al., 2013), but has not yet been used to infer species relationships in the Hieraciinae. The correspondence of cytogenetically employed 45S and 5S rDNA probes with highly variable sequences contained in these regions allows comparing phylogenetic trees of closely related species with the number and localization of the corresponding loci on chromosomes.

Both rDNAs occur in arrays of hundreds to thousands of copies (Long and Dawid, 1980), which are often homogenized by concerted evolution within individuals and species (Arnheim, 1983; Nieto Feliner and Rosselló, 2007). We found previously that ITS is fairly well homogenized in the Hieraciinae (Fehrer et al., 2007a; Ferreira et al., 2015) whereas ETS frequently retained two or more variants in *Hieracium* (Fehrer et al., 2009). This also applied to many diploids investigated and has been attributed to ancient hybridization between lineages or incomplete lineage sorting near the base of the genus. However, some of the ETS variants were found to be homogenized and occasionally shared by other species whereas others were never found as the only variants in any of the species analyzed and were presumed to belong to unknown or extinct lineages. 5S-NTS sequences of two species of *Hieracium* were well homogenized (Zagorski et al., 2020). A few groups of related species were consistently found with different molecular markers (nrDNA, cpDNA, low-copy nuclear genes), but their relationships remained mostly unresolved or were in strong conflict with each other (Krak et al., 2013). So far, each molecular marker applied to *Hieracium* has revealed a particular aspect of the speciation of the genus, but ETS was thought to reflect the evolutionary history best, because it was in concordance with geographic distribution and genome size (Chrtek et al., 2009).

Our initial cytogenetic analyses of *Hieracium* focusing on satellite DNA showed that two species had two 45S rDNA loci and one 5S rDNA locus per haploid genome whereas a third species had three 45S rDNA loci (Belyayev et al., 2018). To assess the variability in the number and position of rDNA loci in *Hieracium*, we extend here the sampling of diploid species and include diploid *Pilosella* and *Andryala* taxa to infer the ancestral pattern in the Hieraciinae. Because, in diploids, the 5S rDNA locus so far always occurred on a chromosome also bearing one of the 45S rDNA loci (Belyayev et al., 2018; Mráz et al., 2019), we ask whether phylogenies based on markers obtained from these regions are congruent or not. We investigate whether the 5S-NTS spacer provides new insights into the diversification of *Hieracium* and related genera, what level of resolution it provides compared to ITS and ETS, how well it is homogenized in the Hieraciinae, if concerted evolution of ITS/ETS and 5S-NTS occurred in the same direction, and how these patterns conform to the number and position of 45S and 5S rDNA loci on chromosomes. We further investigate if cytogenetic patterns are in accordance with the phylogenetic patterns.

## Materials and Methods

### Plant Material

All genera of the Hieraciinae were included in phylogenetic analyses. The little-studied, exclusively American *Hieracium* subgenus *Chionoracium* (Sleumer, 1956) was ignored here because of a lack of material. We also did not include polyploids, because most of the accessions analyzed were found to have allopolyploid origin in *Hieracium* (Krak et al., 2013; Chrtek et al., 2020) and *Pilosella* (Krahulec et al., 2004, 2008; Fehrer et al., 2005, 2007b), and we expected potential confounding effects of reticulation on the organization of the loci (Zagorski et al., 2020).

All major lineages of diploids were represented by 1–3 samples per species, if possible, from different geographic regions. Most diploids of *Hieracium* that had previously shown indications of hybrid origin (showing mixed ETS sequences) were excluded; 18 species included here are representative for all lineages. For *Pilosella* and *Hispidella hispanica*, the same species as in Fehrer et al. (2007a) were sampled (14+1); for some *Pilosella* species, additional accessions were included. *Andryala* was represented by five species, two of which consistently formed long basal branches and another three belonged to the major radiation of the genus (Ferreira et al., 2015; Zahradníček et al., 2018).

Sampling for cytogenetic investigations was as far as possible based on the same individuals that were sequenced; if this was not feasible (herbarium specimens, lack of good metaphases, plants perished), a sample from the same population was sequenced or a larger geographic range was covered by several accessions. Cytogenetic analyses were carried out for a subset of species representing the major lineages; whenever available, more than one individual per species was included. Altogether, 64 samples of 38 species were sequenced, and 29 samples of 18 species were analyzed by fluorescence *in situ* hybridization (FISH). A list of species is provided in Table 1. Details about sample origins and voucher information are included in Supplementary Table 1.

**TABLE 1:** Samples used in this study, their origin, GenBank accession numbers and results from cytogenetic analyses.

Species	Identifier	Origin^4^	GenBank accession numbers			FISH
			
			ETS	ITS	5S-NTS	rDNA loci 5S/45S
*Hieracium alpinum*^1^	alp.Ukr	Ukraine: Polonina Breskulska ridge	EU821408, EU867634	AJ633429	MW328890	
	H63-15-15	Ukraine: Mt. Bliznitsya	MW328990-91	MW325251-52	MW328939	2/4
	H63-30-7	Ukraine: Mt. Bliznitsya	n.d.	n.d.	n.d.	2/4
*H. eriophorum*	1221/1	France: dépt. Landes	EU821409, EU867639	MW315935	MW328891	
	1222/2	France: dépt. Landes	EU867640-41	MW315936, MW587333	n.d.	
	Bis11b	France: Biscarrosse-Plage	MW328992-93	MW325253-54	MW328940	2/4
*H. intybaceum*^2^	inb.Kaer	Austria: Turracher Höhe	EU867568, EU821370	AJ633426, KM372113	MW328892	
	1531/8	Austria: Arlbergpass	MW328994-95	MW325255-56	MW328941-42, MN784131	2/4^5^
	6/14/25	France: Col du Petit Saint-Bernard	MW328996-97	MW325257-58	MW328943-44, MN784130	2/4^5^
*H. kittanae*	1228/2	Bulgaria: central Rhodope Mts	EU821400, EU867622,	MW315937, MW587334	MW328893,	
			MW328998-99	MW325259-60	MW328945-46	
*H. laniferum*	lanif2	Spain: la Sénia	MK523499, MW591759	MW315938, MW587335	MW328894	
*H. lucidum*	H. lucidum	Italy: Sicily, distr. Palermo	EU867592-93	MW315939, MW587336	MW328895	
	Hluc_1-1-2	Italy: Sicily, Mt. Gallo	MW329000-01	MW325261-62	MW328947-48	*/6
*H. petrovae*	1229	Bulgaria: central Rhodope Mts	EU821403, EU867625, MW328989	MW325265, MW587337	MW328949	2/4
*H. plumulosum*	1218/2	Montenegro: canyon of Mrtvica river	FJ858097, FJ858105, FJ858108, FJ858110, MW329002-03	MW325263-64, MW315940	MW328950	
*H. pojoritense*	PM2012	Romania: Pojorita	MW328988	MW325266	MW328951-52	2/4
	poi.Rom.1	Romania: Pojorita	EU867635-36, MK523506-07	AJ633412, MW587338	n.d.	
*H. porrifolium*	1052/9	Austria: Carinthia, Karawanken Mts	EU821407, EU867631	MW315941, MW587339	MW328896	
	Hpor_1-14-2	Slovenia: Podljubelj	MW329004-05	MW325267-68	MW328953-54	
	Hpor_1-14-1	Slovenia: Podljubelj	n.d.	n.d.	n.d.	2/*
	H1463	Slovenia: Julijske Alpe, Spodnja Trenta	n.d.	n.d.	n.d.	*/4
*H. prenanthoides*	1252	France: La Grave	EU821377, EU867579	MW315942, MW587340	MW328897	
	JC1513-3	France: Villarodin & Modane	n.d.	n.d.	n.d.	*/6^6^
	pre_6/5/5	Italy: Claviere	MW329006-07	MW325269-70	MW328955-56, MN784129	2/6^5^
	pre_6/5/2	Italy: Claviere	n.d.	n.d.	n.d.	2/6^6^
	pre_6/8/5	Italy: Claviere	MW329008-09	MW325271-72	MW328957, MN784128	2/6^5^
	pre_6/4/5	Italy: Claviere	n.d.	n.d.	n.d.	2/6
*H. recoderi*	1174/4	Spain: Catalunya, prov. Barce	EU821386, EU867603	MW315943, MW587341	MW328898	
*H. sparsum*	1251/1	Bulgaria: Sofia, Vitoša Mts	EU821404, EU867626	MW315944. MW587342	n.d.	
	spa.sst.2	Bulgaria: Pirin Mts, Vihren	EU867627-28	AJ633431, MW587343	MW328899	
	spa1611/5	Bulgaria: Pirin Mts, Vihren	MW329010-11	MW325273-74	MW328958-59	2/6
	spa1611/6	Bulgaria: Pirin Mts, Vihren	n.d.	n.d.	n.d.	2/6
	PM2099	Bulgaria: Rila Mts, Maljovica	n.d.	n.d.	n.d.	3/6
	PM2102	Bulgaria: Rila Mts, Maljovica	n.d.	n.d.	n.d.	2/6
*H. stelligerum*	1233/1	France: Vallon Pont d‘Arc	EU821383, EU867597	MW315945, MW587344	MW328900	
	Hstel_3-2-1	France: Thueyts	MW329012-13	MW325275-76	MW328960-61	2/7
*H. tomentosum*	1066/8	France: valley of la Roya	EU821382, EU867596	MW315946, MW587345	MW328901	
*H. transylvanicum*^3^	tra.Boa	Romania: Borşa	EU867570-71	MW315947, MW587346	MW328902	
	1077/7	Ukraine: Oblast Zakarpatska	EU821372, EU867572, MW329014-15	MW587347-48 MW325277-78	MW328962	
	Htrans_2-2-1	Romania: Băile Tuşnad	MW328975, MW591760	MW315948	MW328903	2/4
*H. umbellatum*	1021/1	Poland: Województwo pomorskie	EU821410, EU867642	MW315949, MW587349	MW328904	
	um.AM.1	Germany: Schönau-Berzdorf	EU867643-44	KM372116	MW328905	
	H1617	Czechia: Praha, Troja	MW329016-17	MW325279-80	MW328963-64	2/4
	UMB 8/9/3	Slovakia: Prakovce	MW328976, MW591761	MW315950, MW587350	MW328906	2/3
*H. vranceae*	Hvran_1-1	Romania: Lepşa	MK523515, MW591762	MW315951, MW587351	MW328907	
	PM2013	Romania: Lepşa	n.d.	n.d.	n.d.	2/4^7^
*Pilosella alpicola*	pic1141	Slovakia: Vysoké Tatry Mts	MW328977, MW591763	AJ633401	MW328908	
*P. angustifolia*	ang.Fra	France: dép. Hautes-Alpes	MW328978, MW591764	AJ633407	MW328909-13	
*P. argyrocoma*	agy.Gra	Spain: Prov. Granada	KM372001	MW315952	MW328914	
*P. breviscapa*	brc.Bou	France: Lac de Bouillouses	MW328979, MW591765	AJ633393	MW328915	
*P. castellana*	cas.Nev	Spain: Sierra Nevada	MW328980, MW591766	AJ633392	MW328916	
*P. cymosa*	cym.12/4	Czechia: Louny	MW328981, MW591767	AJ633398	MW328917	
*P. echioides*	H1701/2	Czechia: Praha-Čimice	MW329018-19	MW325287-88	MW328965	2/4
*P. hoppeana*	H1702/1	Austria: Carinthia, Hohe Tauern	MW329020-21	MW325281-82	MW328966-67	2/4
*P. lactucella*	lac.Jon.1	Germany: Oberlausitz, Jonsdorf	KM372002	AJ633389	MW328918	
	lac.Neu.2	Germany: Erzgebirge, Neuwernsdorf	MW329022-23	MW325283-84	MW328968-69	
	Zebra	Czechia: Světlá nad Sázavou	n.d.	n.d.	n.d.	2/4
*P. onegensis*	caeb.Jbo.2	Czechia: Krkonoše Mts	MW328982, MW591768	AJ633396	MW328919	
	H1704	Czechia: Krkonoše Mts, distr. Trutnov	MW329024-25	MW325285-86	MW328970-71	2/4
*P. pavichii*	pav.Oly	Greece: Mt. Olympos	MW328983, MW591769	AJ633400	MW328920	
*P. peleteriana*	pel.Wal	Switzerland: Kanton Wallis	MW328984, MW591770	AJ633504	MW328921	
*P. pseudopilosella*	pse.Civ	Spain: prov. Civdad Real	MW328985, MW591771	AJ633390	MW328922	
*P. vahlii*	vah.Sor	Spain: prov. Soria	MW328986, MW591772	AJ633394	MW328923	
*Hispidella hispanica*	His.his.2	Spain: Sierra de Guadarrama	EU821365-66	KM372107	MW328924-28	
*Andryala agardhii*	JC 2011/31/1	Spain: Andalusia, Calar del Desabezedo	KM371905-06	KM372009-10	MW328929	
	A.agaJF	Spain: Sierra Nevada	MW328987, MW591773	MW315953	MW328930	
	PM2390	Spain: garden culture, origin unknown	n.d.	n.d.	n.d.	2/4
*A. glandulosa*	A.glan.Mad.1	Portugal: Madeira, Ponta do Pargo	MW329026-27, KM371929-30	MW325289-90, KM372033-34	MW328931	*
	ZF 233	Portugal: Madeira, Seixal	KM371933-34	KM372037-38	MW328932	
*A. integrifolia*	AZ 4	Algeria: Alger, town distr. Le Caroubier	MW329028-29	MW325291-92	MW328972	*
	AZ 3/1	Algeria: Algiers, Kouba town district	KM371941-42	KM372045-46	MW328933	
	JC 26/1	Spain: Andalusia, province Granada	KM371939-40	KM372043-44	MW328934	
*A. laevitomentosa*	Alev18	Romania: Pietrosul Bogolin	MW329030-31	MW325295-96	MW328973-74	*
	E8	Romania: Pietrosul Bogolin	KM371945-46	KM372049-50	MW328935-36	
*A. pinnatifida*	SB T2/1	Spain: Tenerife, Puerto de la Cruz	KM371981-82	KM372086-87	MW328937	
	And.pin.Cer	Spain: La Gomera, El Cercado	KM371971-72, MW329032-33	KM372076-77, MW325293-94	MW328938	

*^1^An additional sample of *H. alpinum* from a Romanian locality shows the same karyotype (Belyayev et al., 2018), and another sample, also from Romania, has a highly similar ETS sequence (Fehrer et al., 2009) as the Ukrainian material.*

*^2^An additional sample of *H. intybaceum* from Italy shows the same karyotype (Belyayev et al., 2018).*

*^3^Based on a broad geographic sampling, *H. transylvanicum* shows two loci of 5S and four loci of 45S rDNA without intraspecific variation (Ilnicki et al., 2010).*

*^4^For details, see Supplementary Table 1.*

*^5^From Chrtek et al., 2020.*

*^6^From Belyayev et al., 2018.*

*^7^From Mráz et al., 2019.*

n.d., not determined.

*, samples were collected late in the year and root tip quality was insufficient for evaluation or no further material available.

### Sanger Sequencing

Sequences of ITS and ETS of Hieraciinae from previous studies (Fehrer et al., 2007a, 2009; Ferreira et al., 2015) were complemented by newly generated sequences of the same samples (mainly ITS for *Hieracium* and ETS for *Pilosella*). 5S-NTS sequences, so far available for only two species of *Hieracium* (Zagorski et al., 2020), were newly generated for all other samples.

PCR amplification and sequencing of ITS was done as described in Fehrer et al. (2007a), sequencing of ETS followed Fehrer et al. (2009), and procedures for 5S-NTS were as in Kaplan et al. (2013). *Pilosella* samples show a tandem repeat structure in the ETS region and could only be sequenced with the reverse primer, otherwise all sequencing was done in both directions to account for polymorphic sites and to obtain full-length sequences. Polymorphic sites were represented by the IUPAC ambiguity codes and maintained if they were clearly visible on both strands and if their relative amounts were similar, i.e., small additional peaks were ignored so as not to introduce too much noise in phylogenetic analyses. If direct sequences were unreadable due to longer indels, the respective samples were cloned as described in Fehrer et al. (2009); five clones per sample were sequenced in one direction. Sequences were submitted to GenBank (accession numbers MW325251–MW325296, MW315935–MW315953, MW328890–MW329033, MW587333–MW587351, and MW591759–MW591773), see also Table 1.

### Genome Skimming Approach

For 20 samples, low-coverage genome sequencing was newly performed. For these, DNA was extracted from fresh or silica-gel dried leaf tissue using the DNeasy Plant Mini Kit (Qiagen, Hilden, Germany). Library preparation and low-coverage Illumina sequencing were performed at GATC Biotech (Konstanz, Germany)/Eurofins Genomics (Ebersberg, Germany) using a standardized protocol that produced 150 bp paired-end reads with an insert size of ∼450 bp. The raw Illumina datasets have been submitted to the European Nucleotide Archive (ENA) under the study no. PRJEB41719. Raw reads were filtered to remove sequences shorter than 120 bp and Illumina adapters using the Trimmomatic v0.39 tool (Bolger et al., 2014) with parameter settings: ILLUMINACLIP:TruSeq3-PE.fa:2:30:10 LEADING:3 TRAILING:3 SLIDINGWINDOW:4:15 MINLEN:120.

In order to retrieve the sequences corresponding to the 45S rDNA and the 5S rDNA loci, we adopted a reference-guided approach with manual correction based on *de novo* contigs. Our workflow began by creating an unrefined *de novo* assembly from the total low-coverage sequences for a single representative of each of the three genera (*H. kittanae*: 1228/2, *A. laevitomentosa*: Alev18, *P. hoppeana*: H1702) using SPAdes v3.14.0 (Bankevich et al., 2012) with default settings. Contigs corresponding to the 45S rDNA and the 5S/5S-NTS rDNA loci were identified using BLAST+ v2.7.1 (Camacho et al., 2009) (blastn -perc_identity 90 -evalue 1E-50 -max_target_seqs 1) against a database of known sequences (*Helianthus annuus* DQ865267.1 for the 5S, *H. prenanthoides* MN784129.1 for the 5S-NTS, *H. alpinum* EU867634.1 for the 45S rDNA). The contigs provided genus specific reference sequences for the subsequent study.

For each sample, we used BLAST+ to obtain all reads matching the appropriate reference sequences (blastn -word_size 18 -perc_identity 90 -qcov_hsp_perc 55 -max_target_seqs 1). Each sample was thus blasted against one reference sequence for the 45S rDNA and one for the 5S/5S-NTS rDNA locus. Each set of matching reads was corrected for Illumina sequencing errors using the correction algorithm of SPAdes (-only-error-correction -k 21,33,55,77 –careful) followed by correction with Karect (Allam et al., 2015) (correct -matchtype = hamming -celltype = diploid) in order to obtain reads for further mappings and assemblies.

Corrected reads that originated from the two focal markers were mapped on the references using bowtie2 v2.3.5.1 (Langmead and Salzberg, 2012) with stringent settings (-very-fast-local), and reads that failed to align were discarded. For each species, we mapped the reads on the appropriate reference sequence that belonged to the same genus. For each sample/reference combination, we generated a consensus sequence from the mapped reads using Kindel v0.4.2 (Constantinides and Robertson, 2017) (–min-depth 10). Mapped reads were *de novo* assembled using SPAdes (–only-assembler -k 21,33,55,77 –careful). The resulting contigs were aligned together with the consensus sequence with MAFFT v7.471 (Katoh and Standley, 2013) (–adjustdirection –auto –addfragments). The consensus sequences were checked for missing indels by visual comparison with the *de novo* contigs and manually corrected in Bioedit v7.3 (Hall, 1999). A visual sanity check of the bam files was performed in Tablet v1.19.09.03 (Milne et al., 2013). The corrected consensuses were used as references for a final read mapping with bowtie (-very-fast-local) that produced bam formatted files.

Phasing was carried out on the bam files in order to separate allelic variants. Each bam file was analyzed with Samtools v1.10 (Li et al., 2009). The obtained mpileup file was further processed with VarScan v2.3.8 (Koboldt et al., 2012) in order to infer valid single nucleotide polymorphisms (SNPs). Valid SNP positions had to be located in regions with good read coverage (at least eight reads), the minimum number of supporting reads at a position to call a SNP was 2, and each read had to show at the position a minimum base quality of 30 in order to be counted (mpileup2cns –min-coverage 8 –min-reads2 2 –min-avg-qual 30). If valid SNPs were present, the phasing was performed with Samtools phase (-A -F -Q 30). The product of Samtools phase consists at most in two bam files that each correspond to a putative allelic variant. When generated, these files were further subjected to a second Samtools and VarScan round of analyses, and in presence of valid SNPs were further phased in order to produce a maximum of four alleles for each sample/marker combination. Putative chimeric alleles identified by Samtools phase were discarded.

ITS and ETS sequences were extracted from contigs of the entire 45S rDNA region. The phased sequences were aligned with Sanger sequenced samples of all sequences of the same species/individual in BioEdit v7.0.9.0 (Hall, 1999), including sequences with all polymorphic sites retained for comparison with the diversity of phased alleles. After inspection of the variation in each alignment, if more than two phased sequences per sample were found, the two most divergent ones accounting for the maximum of alternative character states at variable sites in ITS as well as ETS regions were chosen to represent the sample. Using the same phased sequence allowed to tentatively assign ITS and ETS allelic variants to each other. 5S-NTS sequences were treated in the same way. We use the following terminology for phased alleles: If only a single variant was found in our approach, the allele is referred to as ‘single’. If two variants occurred, they are labeled 0 and 1. Four alleles (found in the second round of phasing) are designated as 0.0, 0.1 (phasing of the first main variant), 1.0 and 1.1 (phasing of the second variant). If only one new allele was retrieved in the second round of phasing (i.e., a total of three), their labels are 0, 1.0, 1.1 or 0.0, 0.1, 1.

### Phylogenetic Analyses

Total alignments of ITS and ETS were produced in BioEdit and at first subjected to separate phylogenetic analyses to see if topologies were congruent and if phased sequences of both regions corresponded to each other (see Allele matching below). Later, ITS and ETS sequences were concatenated and analyzed together; if one of the regions consisted of a single sequence and the other was represented by two variants, this sequence was concatenated with both sequence variants of the other region. Maximum parsimony (MP), Maximum likelihood (ML), and Bayesian analyses (BA) were carried out using PAUP v4.0b10 (Swofford, 2002), IQ-TREE (Nguyen et al., 2015), and MrBayes v3.2.2 (Ronquist and Huelsenbeck, 2003), respectively. Prior to analysis, gaps were coded as additional characters in FastGap v1.2 (Borchsenius, 2009) using the simple method of Simmons and Ochoterena (2000).

Maximum parsimony analyses were computed as heuristic searches with 100 random addition sequence replicates and TBR branch swapping, saving no more than 100 trees with length greater than or equal to 1 per replicate, automatically increasing the maximum number of trees saved. Bootstrapping was done with the same settings and 1000 replicates, but without branch swapping. For ML and BA, sequence and gap data were treated as separate partitions, applying the GTR2 on the binary partition. Using the ModelFinder (Kalyaanamoorthy et al., 2017) tool of IQ-TREE, the best fitting molecular evolutionary models were determined for ML. The standard non-parametric bootstrap was performed in IQ-TREE with 1000 replicates. For BA, the models best fitting the presumed molecular evolution of the respective datasets were determined with Modeltest v3.5 (Posada and Crandall, 1998) under the Akaike Information Criterion. Models found were TVM + Γ (ETS) and GTR + Γ (ITS, 5S-NTS, combined ITS + ETS). The basic model parameters, i.e., gamma distribution of rates among sites and six different substitution rates, were set as priors for each analysis; apart from that, the default settings were used. Chains were computed for 2 million generations, sampling every 1000th tree; all indicators suggested that convergence between the different runs was achieved for all datasets. The first 25% of the trees per run were discarded as burn-in and the remaining trees were summarized.

In order to characterize the cause of discordance within and between datasets we carried out a Quartet Sampling (QS) analysis (Pease et al., 2018) with 1000 replicates, implemented in the quartetsampling software^1^. A QS analysis provides for each branch three complementary measures: (1) The Quartet Concordance (QC) score that quantifies the support among the three possible resolutions of four taxa. (2) The Quartet Differential (QD) score that measures the disparity between the sampled proportions of the two discordant topologies. QD is only applicable to branches where resampling produces alternative topologies to the input tree. (3) The Quartet Informativeness (QI) score quantifies the proportion of replicates where the best-likelihood quartet has a likelihood score that exceeds the score of the second best quartet. Therefore, these three measures provide an overview of the structure of the topological conflict distinguishing between uninformative branches (signaled by QI) and the branches characterized by conflicting information (QC and QD).

### Allele Matching

We define ‘allele phasing’ as the process of grouping reads according to their shared polymorphisms in order to reconstruct the sequence variants they originate from. Contrary to haplotype phasing, which aims at separating the alleles of the same gene located on different homologous chromosomes, the phasing process in our case groups reads that derive from the same homogenized variants. As a consequence, each phased sequence (termed ‘allele’ here for simplicity) does not necessarily match a single genomic unit, but might represent a majority consensus of several units.

As described above, we mapped the reads from each marker to a single reference sequence and separated the alleles during phasing. However, due to the high level of conservation in the intervening 18S region, there were no polymorphisms located between the ITS and ETS domains that would allow connecting the two regions into a single allele using overlapping pair-reads. Consequently, the phasing could lead to *in silico* recombined 45S alleles where the ITS and ETS regions would not share a common history. Furthermore, because of allele loss, unsampled loci and differential rates of homogenization, we observed a frequent unbalance between the number of ITS and ETS alleles retrieved in a given sample. In order to perform cogent comparisons between the pair of trees derived from the two markers, we designed an algorithm that returns for each accession the best allelic combinations between ITS and ETS sequences. The objective function used to assess the combinations is the distance between the two trees after swapping the leaves’ labels so that they correspond to the selected combination. During the optimization phase, the algorithm searches for the combinations that produce the most similar trees which correspond to the shortest distance between the two trees. The algorithm pre-processes the trees by pruning them to the accessions they have in common, and in each tree, it collapses sister alleles to a single branch whose length corresponds to one of the alleles. Clades made up exclusively of alleles from the same accession are reduced into a single branch that is set to the length of the most basal allele. As coalescent groups of alleles do not provide information for selecting an optimal solution, their removal reduces the combinatorial load. Because the search space grows exponentially with the number of alleles, the number of possible combinations could prove prohibitive in the case of large trees. As a consequence, we designed a heuristic function, which selects the set of the most favorable solutions among all possible solutions for further optimization. The function firstly performs a local optimization that selects a set of optimal pairings for each accession, thus reducing the search space. The possible pairings are then combined during a global optimization, which completes an exhaustive comparison of all remaining combinations using all accessions. For the local optimization, it compares the pre-processed ITS and ETS trees derived from each combination using the Robinson–Foulds (Robinson and Foulds, 1981) distance (aka symmetric distance), which only takes into account the tree topology. The algorithm proceeds by randomly assigning the alleles for all the accessions. Then for each focal accession, all possible combinations are tested while retaining the random combination for the non-focal accessions. For each focal accession, the combinations that minimize the distance between the two modified trees are retained. The product of the best local combinations is then evaluated using a modified Robinson-Foulds metric on rooted trees: Each possible split is weighted by the length of the corresponding branch and by the support of the child node connected to the branch, a support lesser than 50% leads to the removal of the associated split from the distance calculation. The algorithm has been implemented in a Python 3 based software that relies on the Dendropy library (Sukumaran and Holder, 2010). The novel tool (allele_linker) is available at https://git.sorbus.ibot.cas.cz/allele_linker/allele_linker.

### Cytogenetic Experiments and Ancestral State Reconstruction of Locus Numbers

FISH with 45S and 5S rDNA probes was performed as described in Belyayev et al. (2018). The number of 45S and 5S rDNA loci is summarized in Table 1. We refer throughout the manuscript to the total number of loci per diploid genome, corresponding to the number of FISH signals.

For the number of 45S rDNA loci, ancestral state reconstruction was performed based on the combined ITS/ETS tree, either omitting taxa for which locus numbers were unknown or treating them as missing data. ITS is the molecular marker that reflects species relationships in the Hieraciinae best, in accordance with morphology and other evidence (Fehrer et al., 2007a). ETS is the closest approximation of the species tree in *Hieracium* as relationships are in keeping with geographic distribution and genome size (Chrtek et al., 2009; Krak et al., 2013). The combined tree is therefore, despite a lack of resolution in some parts, the best estimate of the species tree. Besides, it is interesting to reconstruct the evolution of 45S rDNA locus numbers on a tree that is based on sequences contained in this locus. Evolution of 5S rDNA locus numbers was not investigated, because they were uniform except for a single sample (see below).

We performed a maximum likelihood reconstruction of ancestral states as a function of stochastic character mapping (SCM) (Huelsenbeck et al., 2003) in R v4.0.3 (R Core Team, 2020). The {phytools} package v0.7-70 (Revell, 2012) was used to project the number of 45S rDNA loci onto the ML tree. The tree was mid-point rooted. It was time-calibrated using the semiparametric penalized likelihood method implemented in the chronopl function of the {ape} package v5.4-1 with a smoothing parameter of 1 (Sanderson, 2002; Paradis et al., 2004). The three usual transition models (ER – equal rates model; SYM – symmetrical model; ARD – all-rates-different model) were compared by computing their corrected Akaike information criterion (AICc) scores. The best-fitting model for character transformation was the ER model (see Supplementary Table 2). Several other custom models that ordered and/or oriented the state transitions were also tested; as they produced identical state reconstructions as the ER model, they will not be further discussed. The character state for specimens that lack a locus count was treated as missing. We reconstructed all changes across the tree based on transitions between the states at each node using the fitDiscrete function of {geiger} package v2.0.7 (Harmon et al., 2008) and mapped them on the ultrametric tree. The magnitude of phylogenetic signal contained in 45S rDNA loci data was evaluated after pruning terminal branches that harbored leaves without locus count. The signal was assessed with Blomberg’s *K* statistics (Blomberg et al., 2003) using the phylosignal function from the {picante} package v1.8.2 (Kembel et al., 2010) and with Pagel’s λ (Pagel, 1994) using the fitDiscrete function with the ER model. Pagel’s λ was computed with 1000 iterations for the pruned tree and for a rescaled tree (no signal model) where all branches were collapsed into a single polytomy. The strength of the phylogenetic signal contained in the locus data was evaluated by comparing the AICc scores for both models.

## Results

### Comparison of Genome Skimming and Sanger Sequencing

Individual alignments for each species showed that polymorphic sites inferred from direct sequencing corresponded very well to the resolved character states of the phased alleles (not shown). In ITS, ETS, and 5S-NTS trees, the position of phased sequences from genome skimming was compared with Sanger sequenced samples of the same species or individual, the latter represented by either major (usually partly polymorphic) or, in some cases (divergent variants), cloned sequences.

**FIGURE 1:**
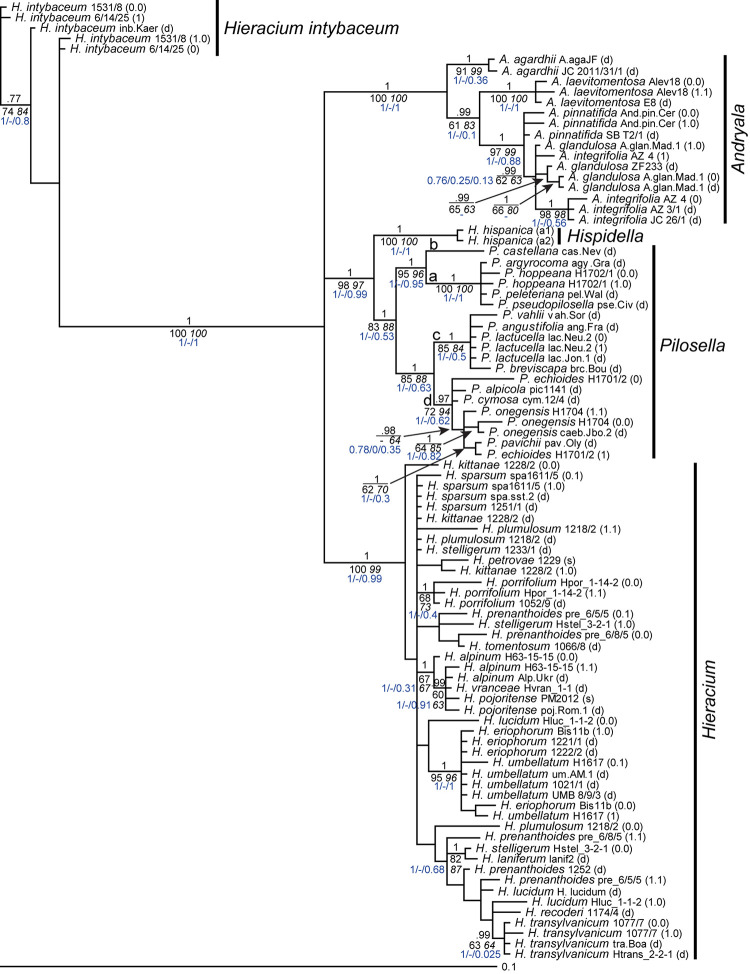
Phylogenetic analysis of the Hieraciinae based on the ITS region. The Bayesian consensus tree is shown with posterior probabilities (pp) above branches and boostrap support (bs) from MP (regular) and ML (italics) analyses below branches. Values are only shown if pp was > 0.94 or bs > 70%. Below the support values, Quartet Concordance/Quartet Differential/Quartet Informativeness scores for 1000 replicates of the full alignment are displayed (in blue). Phased alleles are indicated behind accession labels as 0.0, 0.1, 1.0, 1.1., 0, 1, and s (single); d, direct sequence; a1/a2, two alleles of *Hispidella* occur in ETS sequences, and ITS sequences were duplicated here. a-d, main lineages of *Pilosella*. Accession labels correspond to Table 1.

**FIGURE 2:**
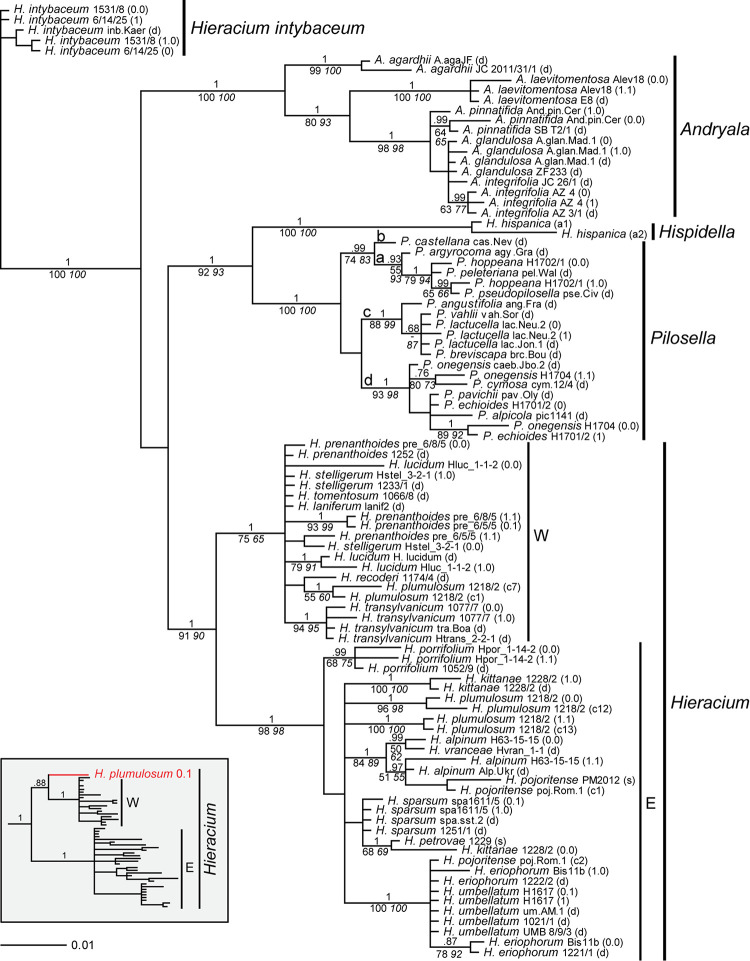
Phylogenetic analysis of the Hieraciinae based on the ETS region. The Bayesian consensus tree is shown with posterior probabilities (pp) above branches and boostrap support (bs) from MP (regular) and ML (italics) analyses below branches. Values are only shown if pp was > 0.94 or bs > 70%. Below the support values, Quartet Concordance/Quartet Differential/Quartet Informativeness scores for 1000 replicates of the full alignment are displayed (in blue). Phased alleles are indicated behind accession labels as 0.0, 0.1, 1.0, 1.1., 0, 1, and s (single); d, direct sequence; a1/a2, two alleles of *Hispidella* (minor and major sequence inferred from direct sequencing); c, cloned sequence. W, E, western and eastern European clades of *Hieracium*. a-d, main lineages of *Pilosella*. Accession labels correspond to Table 1. The inset shows the position of a recombinant phased allele of *H. plumulosum*.

Only in a few cases (*Andryala laevitomentosa*, *Hieracium intybaceum*, *H. porrifolium*, and *H. transylvanicum*), phased alleles and direct sequences of the same species were coalescent. In the ITS tree (Figure 1), phased alleles of several samples (*Pilosella echioides*, *A. integrifolia*, *H. lucidum*, *H. stelligerum* and two accessions of *H. prenanthoides*) were more divergent from each other than different species in their respective clades. Direct sequences of the same sample or species either clustered with one of the phased sequences (*P. onegensis*, *H. alpinum*) or occupied intermediate or basal positions (*H. intybaceum*, *H. kittanae*, *H. lucidum*, *A. pinnatifida*). The same was true for the ETS tree (Figure 2): Divergent alleles (phased sequences) that were more similar to other species occurred (in *P. echioides*, *P. onegensis*, *P. hoppeana*, *H. alpinum*, and *H. kittanae*), intermediate or basal positions of direct sequences were assumed by some species (*H. intybaceum*, *H. alpinum*, and *H. prenanthoides*), or direct sequences clustered with one of the phased alleles (in *A. pinnatifida*, *H. kittanae*, and *H. lucidum*). Worth mentioning is *H. plumulosum*, which was shown to possess four ETS variants, two occurring in the western European clade and two in the eastern European clade (Fehrer et al., 2009). The eastern variants were found among the phased alleles and clustered with two representative clones (12 and 13, the latter clone was recombinant, therefore, only the unique part of this sequence was included in the analyses) whereas the western types (represented by clones 1 and 7) were not retrieved, but only a phased sequence that was recombinant with western clade sequences was detected (Figure 2, inset).

Furthermore, it is likely that phased alleles of ITS and ETS were recombined during the mapping. For example, the ETS sequence of *H. kittanae* 0.0, but the ITS sequence of *H. kittanae* 1.0 clustered with *H. petrovae*. Likewise, *P. echioides* 1 clustered with *P. pavichii* in the ITS tree, but with *P. onegensis* 0.0 in the ETS tree. Therefore, before concatenating ITS and ETS sequences for combined analyses, we tested for *in silico* recombination of the phased alleles using allele_linker (see below).

**FIGURE 3:**
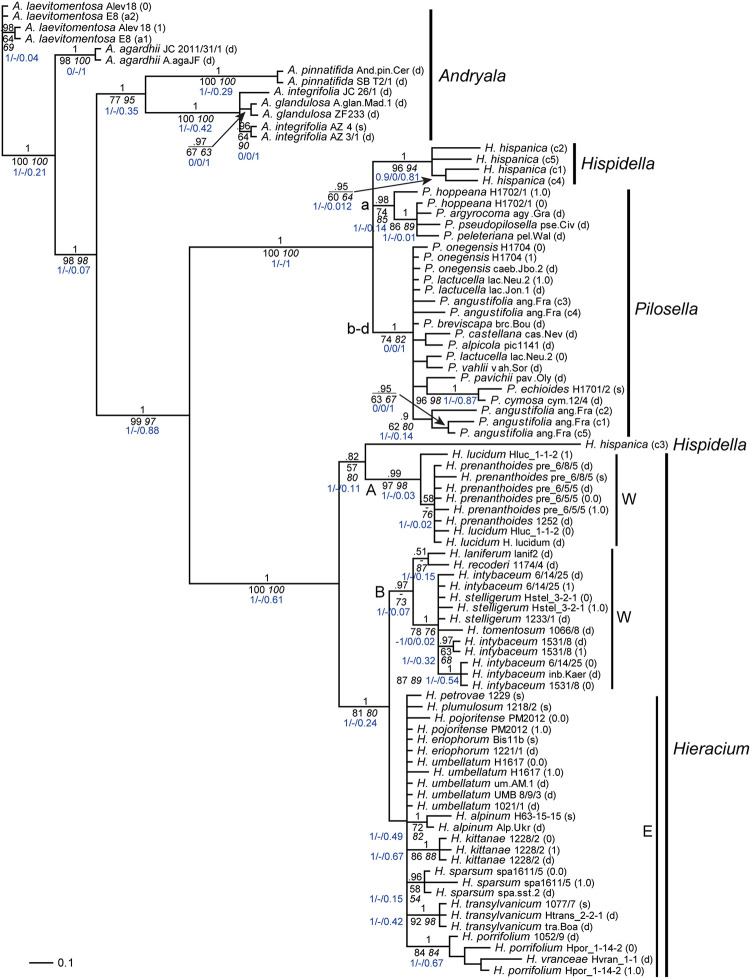
Phylogenetic analysis of the Hieraciinae based on the 5S-NTS region. The Bayesian consensus tree is shown with posterior probabilities (pp) above branches and boostrap support (bs) from MP (regular) and ML (italics) analyses below branches. Values are only shown if pp was > 0.94 or bs > 70%. Below the support values, Quartet Concordance/Quartet Differential/Quartet Informativeness scores for 1000 replicates of the full alignment are displayed (in blue). Phased alleles are indicated behind accession labels as 0.0, 0.1, 1.0, 1.1., 0, 1, and s (single); d, direct sequence; c, cloned sequence; a1/a2, variants of *A. laevitomentosa* E8 differing by a single substitution. W, E, western and eastern European clades of *Hieracium*; A, B, two clades of *Hieracium* with western European origin. a-d, main lineages of *Pilosella*. Accession labels correspond to Table 1.

5S-NTS alleles were generally less divergent than ITS or ETS alleles (Table 2); often, only a single variant was found in sequences retrieved from genome skimming, and direct sequences were fairly homogenous as well. Species in which phased alleles and direct sequences formed coalescent groups were *A. laevitomentosa*, *H. alpinum*, *H. sparsum*, *H. kittanae*, and *H. transylvanicum* (Figure 3). In *P. hoppeana*, one of the phased alleles was more similar to other *Pilosella* species than to the second allele of the same individual, the same held for *H. lucidum*. One sample of *A. integrifolia* (JC 26/1) did not group with other samples of the same species. *Pilosella angustifolia* and *Hispidella hispanica* showed large indels in direct sequencing. Three of the cloned sequences of *P. angustifolia* grouped together, the other two occurred in unresolved positions among other *Pilosella* species. Four cloned sequences of *Hispidella* formed a well-supported branch, but another cloned sequence inserted near the base of genus *Hieracium*.

**TABLE 2:** Number of polymorphic sites in direct sequences of Hieraciinae and diversity of phased or cloned sequences.

Species	Identifier	No. of polymorphic sites (d) or substitutions (ph, c)				No. of 45S
		
		ETS	ITS	ETS + ITS	5S-NTS	rDNA loci
*Hieracium alpinum*	alp.Ukr	3/4 (d)	3/3 (d)	6/7 (d)	0 (d)	
	H63-15-15	8 (ph)	2 (ph)	10 (ph)	0 (ph)	4
*H. eriophorum*	1221/1	5/6 (d)	3/3 (d)	8/9 (d)	7 (d)	
	1222/2	7/8 (d)	0/5 (d)	7/13 (d)	n.d.	
	Bis11b	7 (ph)	5 (ph)	12 (ph)	0 (ph)	4
*H. intybaceum*	inb.Kaer	1/4 (d)	0/8 (d)	1/12 (d)	0 (d)	
	1531/8	1 (ph)	6 (ph)	7 (ph)	2 (ph)	4
	6/14/25	1 (ph)	6 (ph)	7 (ph)	1 (ph)	4
*H. kittanae*	1228/2	0/15 (d), 17 (ph)	5/9 (d), 9 (ph)	5/24 (d), 26 (ph)	1 (d), 1 (ph)	
*H. laniferum*	lanif2	1/2 (d)	1/11 (d)	2/13 (d)	8 (d)	
*H. lucidum*	H. lucidum	1/13 + indel (d)	2/30 (d)	3/43 + indel (d)	3 (d)	
	Hluc_1-1-2	13 + indel (ph)	17 (ph)	30 + indel (ph)	4 (ph)	6
*H. petrovae*	1229	1/7 (d), 0 (ph)	0/10 (d), 0 (ph)	1/17 (d), 0 (ph)	0 (ph)	4
*H. plumulosum*	1218/2	35 + indel (ph + c)	21 (d), 19 (ph)	56^2^ + indel	0 (ph)	
*H. pojoritense*	PM2012	0 (ph)	0 (ph)	0 (ph)	2 (ph)	4
	poi.Rom.1	1/26 (d), 11 (c)	2/21 + indel (d)	3/48 + indel (d)	n.d.	
*H. porrifolium*	1052/9	5/6 (d)	2/3 (d)	7/9 (d)	2 (d)	4*
	Hpor_1-14-2	7 (ph)	7 (ph)	14 (ph)	3 (ph)	4*
*H. prenanthoides*	1252	3/8 (d)	18/23 (d)	21/31 (d)	4 (d)	
	pre_6/5/5	8 (ph)	13 (ph)	21 (ph)	0 (d), 1 (ph)	6
	pre_6/8/5	6 (ph)	15 (ph)	21 (ph)	0 (d), 0 (ph)	6
*H. recoderi*	1174/4	2/5 (d)	1/4 (d)	3/9 (d)	4 (d)	
*H. sparsum*	1251/1	3/12 (d)	8/9 + indel (d)	11/21 + indel (d)	n.d.	
	spa.sst.2	1/12 (d)	4/6 (d)	5/18 (d)	0 (d)	
	spa1611/5	1 (ph)	4 (ph)	5 (ph)	3 (ph)	6
*H. stelligerum*	1233/1	1/3 (d)	18/21 (d)	19/24 (d)	1 (d)	
	Hstel_3-2-1	2 (ph)	13 (ph)	15 (ph)	1 (ph)	7
*H. tomentosum*	1066/8	0/6 (d)	1/15 (d)	1/21 (d)	1 (d)	
*H. transylvanicum*	tra.Boa	0/5 (d)	0/2 (d)	0/7 (d)	0 (d)	
	1077/7	0/7 (d), 3 (ph)	0/3 (d), 2 (ph)	0/10 (d), 5 (ph)	0 (ph)	
	Htrans_2-2-1	0/3 (d)	1/1 (d)	1/4 (d)	0 (d)	4
*H. umbellatum*	1021/1	2/5 (d)	7/8 (d)	9/13 (d)	0 (d)	
	um.AM.1	0/5 (d)	6/6 (d)	6/11 (d)	1 (d)	
	H1617	0 (ph)	6 (ph)	6 (ph)	2 (ph)	4
	UMB 8/9/3	0/4 (d)	6/8 (d)	6/12 (d)	0 (d)	3
*H. vranceae*	Hvran_1-1	0/1 (d)	2/4 + indel (d)	2/5 + indel (d)	1 (d)	4*
*Pilosella alpicola*	pic1141	0/3 (d)	0/n.a. (d)	0/ ≥ 3 (d)	0 (d)	
*P. angustifolia*	ang.Fra	2/6 + indel (d)	0/n.a. (d)	2/ ≥ 6 + indel (d)	10 + 3 indels (c)	
*P. argyrocoma*	agy.Gra	6/6 (d)	7/7 (d)	13/13 (d)	0 (d)	
*P. breviscapa*	brc.Bou	4/5 (d)	0/n.a. (d)	4/ ≥ 5 (d)	1 (d)	
*P. castellana*	cas.Nev	4/6 (d)	0/n.a. (d)	4/ ≥ 6 (d)	1 (d)	
*P. cymosa*	cym.12/4	3/10 (d)	0/n.a. (d)	3/ ≥ 10 (d)	6 (d)	
*P. echioides*	H1701/2	3 (ph)	10 (ph)	13 (ph)	0 (ph)	4
*P. hoppeana*	H1702/1	3 (ph)	3 (ph)	6 (ph)	4 (ph)	4
*P. lactucella*	lac.Jon.1	1/1 (d)	0/n.a. (d)	1/ ≥ 1 (d)	2 (d)	4*
	lac.Neu.2	1 (ph)	0 (ph)	1 (ph)	2 (ph)	4*
*P. onegensis*	caeb.Jbo.2	6/8 (d)	2/n.a. (d)	8/ ≥ 10 (d)	1 (d)	
	H1704	12 (ph)	5 (ph)	17 (ph)	1 (ph)	4
*P. pavichii*	pav.Oly	0/5 (d)	0/n.a. (d)	0/ ≥ 5 (d)	3 (d)	
*P. peleteriana*	pel.Wal	0/2 (d)	1/n.a. (d)	1/ ≥ 3 (d)	0 (d)	
*P. pseudopilosella*	pse.Civ	1/3 (d)	0/n.a. (d)	1/ ≥ 3 (d)	0 (d)	
*P. vahlii*	vah.Sor	2/4 (d)	0/n.a. (d)	2/ ≥ 4 (d)	0 (d)	
*Hispidella hispanica*	His.his.2	6 + indel (d)^1^	1/1 (d)	7 + indel (d)	53 + 4 indels (c)	
*Andryala agardhii*	JC 2011/31/1	0/10 (d)	0/13 (d)	0/23 (d)	0 (d)	4*
	A.agaJF	6/9 (d)	11/11 (d)	17/20 (d)	1 (d)	4*
*A. glandulosa*	A.glan.Mad.1	0/2 (d), 1 (ph)	0/2 (d), 2 (ph)	0/4 (d), 3 (ph)	0 (d)	
	ZF 233	0/1 (d)	1/3 (d)	1/4 (d)	0 (d)	
*A. integrifolia*	AZ 4	1 (ph)	8 (ph)	9 (ph)	0 (ph)	
	AZ 3/1	1/2 (d)	0/11 (d)	1/13 (d)	0 (d)	
	JC 26/1	0/1 (d)	1/1 (d)	1/2 (d)	0 (d)	
*A. laevitomentosa*	Alev18	3 (ph)	3 (ph)	6 (ph)	1 (ph)	
	E8	1/4 (d)	2/3 (d)	3/7 (d)	1 (d)	
*A. pinnatifida*	SB T2/1	0/1 (d)	1/4 (d)	1/5 (d)	0 (d)	
	And.pin.Cer	0/6 (d), 5 (ph)	1/3 (d), 4 (ph)	1/9 (d), 9 (ph)	0 (d)	

*For direct ITS and ETS sequences, the number of polymorphic sites is provided for the major sequence/for the sequence including all polymorphisms. Sequence reads of 5S-NTS did in many cases not allow reliable identification of smaller additional peaks due to polynucleotide runs and high GC content. However, very low numbers of substitutions between phased alleles of the same sample indicate that there is indeed not much hidden variation.*

d, direct sequence; ph, phased alleles; c, cloned sequences; n.d., not determined; n.a., number of all polymorphic sites not available (sequences provided by a collaborator).

*, locus number determined for another individual and assigned to the species.

*^1^Major and minor variant inferred from direct sequencing.*

*^2^The maximal number of substitutions is given.*

*The combined variation of ITS and ETS sequences and the number of corresponding 45S rDNA loci are shown. All sequenced samples have two loci of 5S rDNA.*

### Comparison of Tree Topologies Based on ITS and ETS

Generally, species relationships based on both rDNA regions were fairly similar and in agreement with previous findings (Fehrer et al., 2007a, 2009; Ferreira et al., 2015; Mráz et al., 2019). These concern the outgroup position of *Hieracium intybaceum*, the sister relationship of *Hispidella* and *Pilosella*, a main clade of all *Andryala* species except *A. agardhii* and *A. laevitomentosa*, four lineages within *Pilosella* with identical species compositions, a joint clade of *H. umbellatum* and *H. eriophorum* (with ETS also containing an allele of one sample of *H. pojoritense*). For all these clades, the basal branch displayed a perfect QS score (i.e., 1/-/1), indicative of high data informativeness and no conflict. A clade comprising *H. alpinum*, all other alleles of *H. pojoritense*, and *H. vranceae* was also recovered in both trees, but its basal branch showed slightly less information without conflicting quartets with QS of 1/-/0.91 for the ITS and 1/-/0.69 for the ETS tree. Also, species relationships within *Hieracium* remained largely unresolved with ITS whereas a marked separation into two groups of mainly western or eastern European origin was found with ETS. However, the western clade is poorly supported by MP and ML bootstrap values (75 and 65 respectively) and the QS analysis shows that only a week majority of quartets supports the corresponding branch (QC = 0.062), which can be resolved equally into any of the possible topologies (QD = 0) despite that the data contains a rather high phylogenetic signal (QI = 0.55). According to Pease et al. (2018), this QS configuration is indicative of a rapid radiation or a highly complex conflict.

### Combining ITS and ETS Sequences for Phylogenetic Analysis

To account for recombination in the 18S rDNA, the newly designed software run using the ML trees indicated phased ITS and ETS sequences of the following samples should be swapped: *P. echioides*, *H. kittanae*, *H. stelligerum*, *H. eriophorum*, and *H. prenanthoides* pre_6/5/5. The algorithm further suggested to swap alleles of both *H. intybaceum* accessions, which is equivalent to changing none of them and was therefore dismissed, and to swap the alleles of *H. porrifolium.* Because these formed a well-supported branch together with the direct sequence of another accession of the same species in BA of ITS and ETS trees without further resolution of their relationships, swapping of the alleles is irrelevant. Equivocal results (no preference for swapping or not swapping of alleles) were obtained for *H. alpinum*, *H. prenanthoides* pre_6/8/5, *A. integrifolia* and *H. umbellatum*. Alleles of the latter three were not swapped, because nothing indicated that either solution was better for the combined analysis, however, *H. alpinum* allele 0.0 grouped with *H. vranceae* in the ETS tree whereas allele 1.1 grouped with *H. vranceae* in the ITS tree with high support in BA. Therefore, swapping of *H. alpinum* alleles was performed as well.

After swapping of these six allele pairs, combined analyses were performed (omitting *H. plumulosum* and clone 2 of *H. pojoritense* poi.Rom.1). Expectedly, the resolution of the tree was enhanced (Supplementary Figure 1) as indicated by higher support values, but no additional species relationships compared to individual analyses of ITS and ETS sequences were found. It is noteworthy that combining the two markers greatly boosts the phylogenetic signal for the western clade in *Hieracium*, which leads to higher MP and ML bootstrap support values (95 and 88 respectively) and a near perfect QS score (1/-/0.67) clear of topological conflict.

### Phylogenetic Analysis Based on the 5S-NTS and Comparison With ITS/ETS Tree Topology

The most striking difference of the 5S-NTS tree (Figure 3) compared to those based on ITS and ETS was the position of *Hieracium intybaceum*, which did not form an outgroup to the rest of the Hieraciinae, but clustered with several *Hieracium* species of western European origin. The high support values for the western European B clade containing *H. intybaceum* in the former tree and the taxon’s outgroup position of *H. intybaceum* in the latter tree point toward a genuine phylogenetic signal as the cause of this topological conflict. QS scores corroborate this conclusion by displaying a perfect score for the branch leading to the ingroup in the ITS/ETS tree (Supplementary Figure 1). Although several branches within the B clade possess low QI scores (0.07) in the 5S-NTS tree, no alternative topology is supported (QC of 1 and QD undefined) for any branch between the outgroup and the B clade.

Further differences of 5S-NTS tree topology compared to ITS/ETS topology were as follows: With *Pilosella*, only one of the four lineages found previously was retrieved (a); the other three (b–d) formed a single clade with mostly unresolved species relationships. The majority of cloned *Hispidella* sequences was sister to both *Pilosella* lineages. It is tantalizing to interpret the position of *H. hispanica* (c3) in the 5S-NTS tree as caused by a low phylogenetic signal (QI score of 0.11 for the branch grouping the sequence with the A clade). However, as no alternative topology is supported on any branch leading to this terminal (QC of 1 and QD undefined), the non-monophyly of *Hispidella* in this tree seems genuine and does not stem from an artifact of the phylogenetic reconstruction, but requires a biological explanation.

With *Andryala*, the only difference concerned a clear separation of *A. pinnatifida* from *A. glandulosa* and *A. integrifolia*. With *Hieracium*, species of western European origin were separated into two clusters, one of them (A, consisting of *H. lucidum* and *H. prenanthoides*) was sister to one cloned sequence of *Hispidella*, the other cluster (B, consisting of *H. stelligerum*, *H. tomentosum*, *H. intybaceum* and the Pyrenean species *H. laniferum* and *H. recoderi*) formed a well-supported branch together with all species of eastern European origin. The eastern lineage was still recognizable, however, poorly resolved. Finally, *Hieracium vranceae* nested among sequences of *H. porrifolium*, and *H. transylvanicum* belonged to the eastern European species of *Hieracium.* In these two cases, within each tree the branches leading to the sequences show a high level of informativeness (QI above 0.5) and no conflict between the trees and the data used to generate them (QC of 1 and QD undefined), which indicates that no alternative evolutionary history is favored by any of the branches.

### Organization of 45S and 5S rDNA in Relation to Phylogeny

**FIGURE 4:**
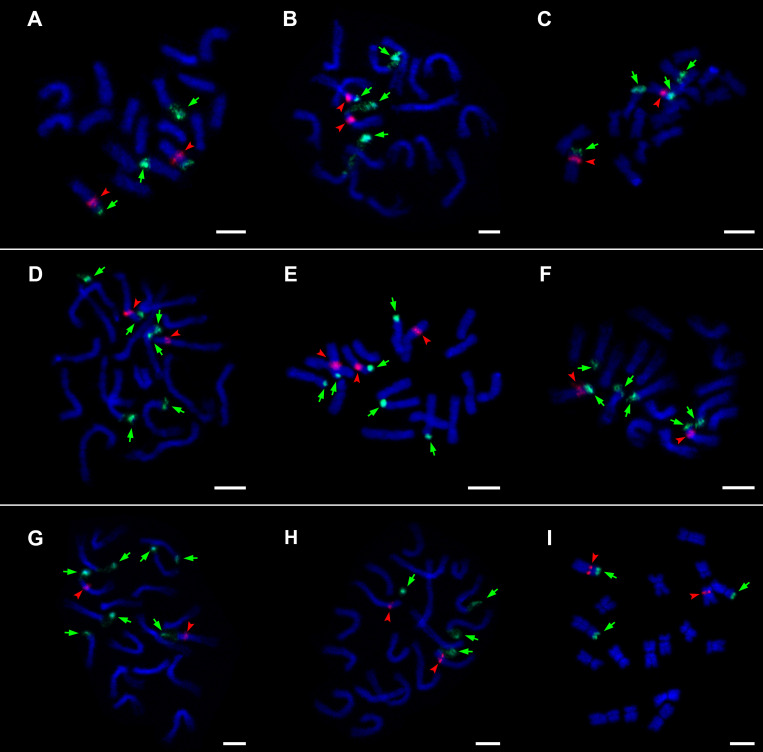
Visualization of 45S rDNA and 5S rDNA loci on metaphase chromosomes. **(A)** *Andryala agardhii* PM2390, **(B)** *Pilosella echioides* H1701/2, **(C)** *P*. *hoppeana* H1702/1, **(D)** *Hieracium sparsum* PM2102, **(E)** *H. sparsum* PM2099, **(F)** *H. sparsum* spa1611/6, **(G)** *H*. *stelligerum* Hstel_3-2-1, **(H)** *H*. *umbellatum* H1617, **(I)** *H*. *umbellatum* UMB 8/9/3. 45S rDNA (green signal and arrows) and 5S rDNA (red signal and arrowheads). Chromosomes were counterstained with DAPI (blue). Scale bars = 5 μm.

45S rDNA loci always occurred in terminal positions and 5S rDNA loci in interstitial positions; the 5S locus was always localized on the same chromosome with one 45S locus. The majority of samples of the three genera showed four loci of 45S and two loci of 5S rDNA per diploid genome (Table 1 and Figure 4). In *Hieracium*, which was investigated in more detail, all analyzed accessions of three species (*H. prenanthoides*, *H. sparsum*, and *H. lucidum*) had six loci of 45S rDNA, *H. stelligerum* (Hstel_3-2-1) had seven loci, and one accession of *H. umbellatum* (UMB 8/9/3) had only three loci. In the latter, the 45S rDNA locus was lost together with a part of the chromosome arm (Figure 4I). Differences in the number of 5S loci were only found in one accession of *H. sparsum* (PM2099) whereas another sample of the same population (PM2102) and two samples of another population showed the usual two loci. No indications for translocations or inversions were observed.

For the combined ITS/ETS tree, the magnitude of the phylogenetic signal associated with the 45S rDNA loci count was measured with Blomberg’s *K* and Pagel’s λ statistics. For both indices, a value close to 0 indicates phylogenetic independence and a value of 1 indicates that species’ traits are distributed as expected under a Brownian motion model of trait evolution. Blomberg’s *K* revealed a moderate phylogenetic signal that was significantly different from zero (0.3519 *P*-value < 0.005); in contrast, Pagel’s λ with a value of 0.7746 indicated an intense signal and strongly rejected the no-signal model (dAICc = 11.0717). This difference in magnitude could stem from the structure of the tree having a differential effect on the ability to accurately measure phylogenetic signal. Our tree contained a number of near zero length internal branches that could hinder Blomberg’s *K* performance whereas they should not affect Pagel’s λ (Münkemüller et al., 2012). In all cases, they clearly indicate that the trait is not randomly distributed, but follows largely the pattern of the phylogeny.

**FIGURE 5:**
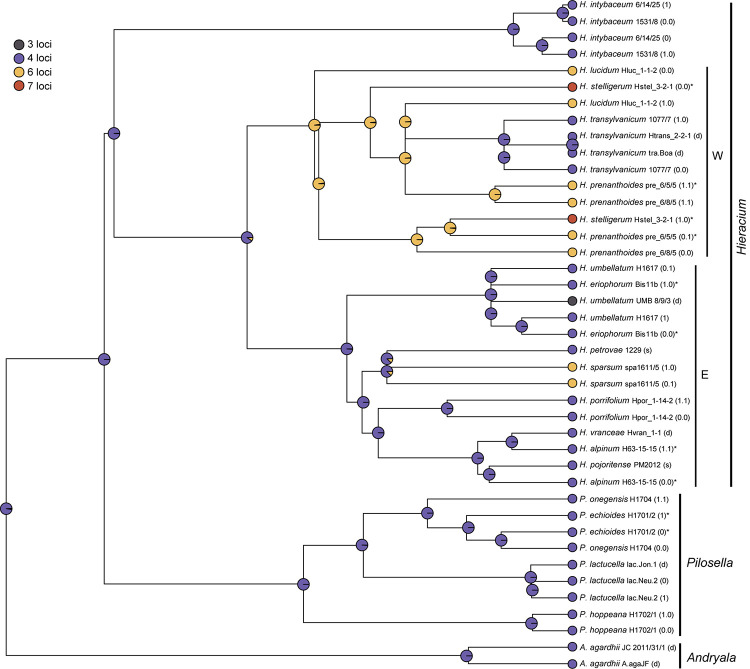
Ancestral character state reconstruction on the maximum likelihood tree based on the combined ITS and ETS sequences using stochastic mapping of 45S rDNA loci. Locus numbers (see Table 1) were assigned to sequences (alleles) of the same individual. For *H. transylvanicum*, all further individuals were assigned 4 loci, because this species does not show intraspecific variation (Ilnicki et al., 2010). For *A. agardhii*, *P. lactucella*, *H. porrifolium*, and *H. vranceae*, for which only cytogenetic data from other individuals were available, locus numbers were assigned to the species. Pies at nodes represent the marginal ancestral states (empirical Bayesian posterior probabilities). Phased alleles are indicated behind accession labels as 0.0, 0.1, 1.0, 1.1., 0, 1, and s (single). Labels correspond to those in the ITS tree (Figure 1); swapped alleles for ETS are marked by asterisks (*). d, direct sequence; W, E, western and eastern European clades of *Hieracium*.

The ancestral number of 45S rDNA loci in Hieraciinae was four whereas all other numbers were derived as they mainly occurred close to the tips (Figure 5 and Supplementary Figure 2). *Hieracium* species of western European origin showed six or seven loci, the latter appeared to be derived from the former. An exception was the state of *H. transylvanicum*, contained in this clade with four loci, but this was considered as an artifact of the placement of this eastern European species in the ‘wrong’ clade (see section Discussion) rather than a secondary reduction of locus numbers. Six locus numbers occurred independently in *H. sparsum*, which belongs to the eastern European lineage, and three loci occurred only in one sample of *H. umbellatum*, which is a derived character state.

## Discussion

### Features and Molecular Evolution of rDNA Sequences

rDNA sequences of many individuals and species of the Hieraciinae were fairly well homogenized for all markers. In these cases, no or only a few polymorphic sites were found in direct sequences, or a single or two fairly similar sequences were retrieved by the genome skimming approach. If more than two alleles were determined for an individual, those from the first round of phasing (0, 1) were, except in *H. plumulosum* (see section “Results”), always more divergent than alleles found in the second round of phasing; these formed pairs of similar alleles (0.0, 0.1 and 1.0, 1.1, respectively). To our knowledge, phasing of rDNA sequences obtained from genome sequencing has not been attempted before, and in fact, even phasing of low-copy genes is rarely being done for phylogenetic inference even though it has been shown that phasing might improve the phylogenetic analysis (Eriksson et al., 2018). We show that results from direct sequencing and cloning are well comparable to the genomic approach in Hieraciinae and provide a new software tool (allele_linker) that allows to assign ITS and ETS alleles for combined analysis. The tool can also be used for pairwise assignment of alleles from two different markers in order to find correspondences between leaves in the trees that are necessary for phylogenetic inferences based on concatenated data, or for genome tree reconstruction where a species tree is built from non-concatenated markers and each allele/paralogue combination represents an evolutionary lineage.

In phylogenetic analyses, inclusion of polymorphic sequences along with phased (or cloned) alleles of the same sample or species resulted in patterns typically observed for ITS and ETS sequences of hybrids or allopolyploids: Polymorphic sequences (analogous to hybrid sequences) end up at or near the base of the clade containing the separated variants, or cluster with one of the variants, or occur in a basal position relative to all other ingroup taxa (Soltis et al., 2008). The latter possibility was not found in our study, probably because polymorphic sequences actually did not belong to hybrids (most diploid samples with mixed sequences that were additive for different lineages were excluded *a priori*), the level of polymorphism was usually low, and the dominant sequence (ignoring small additional peaks) used for phylogeny reconstruction often matched that of one phased allele as should be expected.

Alleles of some species formed coalescent groups, but often, sequences were similar or identical between closely related species indicating very recent divergence of the respective taxa or, maybe less likely, homogenization of rDNA toward the same variant. ITS and ETS sequences were generally more variable within individuals than 5S-NTS sequences (Table 2). This might, at least in part, be due to the higher length of aligned ITS (695 bp) and ETS (574 bp) sequences compared to 5S-NTS (296 bp). Nevertheless, compared to its length, the overall variation in the 5S-NTS was larger than that of both ITS and ETS: 5S-NTS showed 114 parsimony informative characters (38.5%) whereas ITS showed 135 (19.4%) and ETS 137 (23.9%). A much higher proportion of parsimony informative characters in 5S-NTS sequences compared to ITS sequences was also found in *Anemone* (Mlinarec et al., 2012). On the other hand, ITS and ETS together provided more than twice as many parsimony informative characters for phylogenetic analysis. ITS/ETS and 5S-NTS showed different resolution in different parts of the tree as well as several highly incongruent patterns. This indicates that while the 5S locus is always located on the same chromosome with one of the 45S rDNA loci, their sequences evolved independently. Independent evolution of both arrays was also reported in other plants and in animals (Rosato et al., 2015; Araya-Jaime et al., 2020).

Very often, ITS sequences of diploids are well homogenized in plants (Baldwin et al., 1995) as evidenced by their widespread use for building phylogenies despite their well-known drawbacks (Álvarez and Wendel, 2003; Nieto Feliner and Rosselló, 2007). The same holds for the less often used ETS, which is often more variable than the ITS, and the phylogenetic signal from both regions is usually congruent and provides better resolution and higher support in trees (Baldwin and Markos, 1998; Calonje et al., 2009). Both regions are part of the array forming the nucleolar organizer region (Hillis and Dixon, 1991); they associate during interphase, and interlocus homogenization is a common observation where multi-gene families are located in terminal positions on chromosomes (Cronn et al., 1996; Volkov et al., 1999; Rauscher et al., 2004). In contrast, 5S-NTS sequences are usually highly polymorphic within individuals and often exhibit different unit size classes (e.g., Kellogg and Appels, 1995; Besendorfer et al., 2005; Galián et al., 2014; and references therein). For this reason, most research is focused on the molecular patterns of this region, but it is less often used for phylogeny reconstruction (e.g., Lan and Albert, 2011; and references therein). In the Hieraciinae, 5S-NTS sequences are exceptionally well homogenized. As almost all of them possess only one 5S rDNA locus per haploid genome, intralocus homogenization may be more efficient in this case than interlocus homogenization of 45S rDNA. However, Volkov et al. (2007) suggested that concerted evolution generally operates differently in 5S rDNA. Also, other factors such as the number of repeats in an array, the intensity of natural selection and effective population size can play a role (Smith, 1976; Basten and Ohta, 1992; Schlötterer and Tautz, 1994). Lower copy numbers of 5S arrays compared to 45S arrays are often observed (Sastri et al., 1992; Macas et al., 2011), also in *Hieracium* (Zagorski et al., 2020), although a stoichiometric relationship of mature rRNA copies from genes of both loci is required for ribosome biogenesis (Fromont-Racine et al., 2003). Our findings add to the vast literature on differential behavior of unlinked rDNA arrays in plants and animals.

### Phylogenetic Trees Reveal Hybridization and Differential Homogenization of rDNA

In the Hieraciinae, several cases of ancient intergeneric hybridization were found previously based on the discrepancy between chloroplast and nuclear DNA markers (Fehrer et al., 2007a). In all genera, especially within *Hieracium*, massive allele sharing of various molecular markers between species was inferred, and many interspecific relationships remained unresolved (Fehrer et al., 2009; Krak et al., 2013; Ferreira et al., 2015; Mráz et al., 2019; Chrtek et al., 2020). Reasons for these patterns were a lack of divergence in closely related species, incomplete lineage sorting, and hybridization. Phased alleles of ITS and ETS added further complexity at the intra-individual level, and the 5S-NTS, which was investigated for Hieraciinae for the first time, provided novel insights into the intricate evolution of this group. Particular patterns of some species are discussed in the following paragraphs.

In the case of *Hieracium intybaceum*, all nuclear markers employed previously (ITS – Fehrer et al., 2007a; ETS – Fehrer et al., 2009; *sqs* – Krak et al., 2013; *gsh1* – Chrtek et al., 2020) placed the species in an outgroup position. *Hieracium intybaceum* is considered as an ancient intergeneric hybrid involving a parent whose nuclear DNA markers belonged to an extinct taxon (Fehrer et al., 2007a; Krak et al., 2012, 2013; Chrtek et al., 2020). The 5S-NTS is the first nuclear marker that groups *H. intybaceum* with other *Hieracium* species; it occurs in a well-supported cluster with the western European species *H. stelligerum* and *H. tomentosum*, and its sequences are highly similar to those of these species. In contrast, its chloroplast DNA clusters with the eastern European species *Hieracium alpinum*, *H. sparsum*, *H. pojoritense*, and *H. vranceae* (Krak et al., 2013; Mráz et al., 2019). The repetitive part of its genome is highly similar to *Hieracium* species of western as well as eastern European origin (*H. prenanthoides*, *H. umbellatum*; Zagorski et al., 2020), and *H. intybaceum* also shows an abundant, *Hieracium*-specific tandem repeat located in the centromeric regions of all chromosomes (Belyayev et al., 2018). As the species forms many polyploid apomictic hybrids with *Hieracium* species (Zahn, 1921–1923; Chrtek et al., 2020), it was included in this genus despite its markedly different morphology (Zahn, 1921–1923). It shares also the same chromosomal organization of rDNA with the majority of the Hieraciinae, however, concerted evolution of ITS/ETS (45S rDNA loci) and 5S-NTS (5S rDNA loci) operated in opposite directions – toward the extinct parent or toward *Hieracium*, respectively. The fact that it clusters with different lineages of *Hieracium* in chloroplast and 5S-NTS trees may indicate that the hybridization event occurred early, before western and eastern European lineages of *Hieracium* diverged. Alternatively, given the sequence similarity with different contemporary species, *H. intybaceum* may have been introgressed by a second lineage of *Hieracium* after the initial hybridization event. At the intraspecific and intra-individual level, all non-coding rDNA regions are very well homogenized, and indications for its hybrid origin are based on the discrepancies of different molecular markers rather than on mixed sequences of the same markers. Sequences of all other molecular markers as well as AFLP patterns (Zahradníček and Chrtek, 2015) are fairly homogenous across populations indicating a single, however, complex origin of this species.

A further case of homogenization of ITS/ETS and 5S-NTS into different directions concerns *Hieracium transylvanicum*. Intraspecific variation was also very low, and all rDNAs were well homogenized. All alleles of all accessions of this species formed well-supported coalescent groups, but these occurred in the western European clade with ITS/ETS, but among eastern European species with 5S-NTS. The latter placement is in accordance with its geographic origin and genome size (Chrtek et al., 2009). This pattern may be explained by incomplete lineage sorting of western and eastern European alleles of *Hieracium*.

*Hieracium vranceae* is a recently described species of the Carpathians (Mráz et al., 2019). Based on ITS and ETS (and chloroplast DNA), it is most closely related to *H. alpinum* and *H. pojoritense* occurring in the same area. Surprisingly, it formed a well-supported branch together with the southeastern alpine species *H. porrifolium* with the 5S-NTS. This is not a methodological artifact, because its 5S-NTS sequence was unique and well homogenized, but not identical to any of the *H. porrifolium* sequences. In this case, we may observe either hidden introgression or incomplete lineage sorting. A second sample of *H. vranceae* showed two divergent ETS alleles (Mráz et al., 2019), one of which was nearly identical with the sequence of the individual included here, the other occurred in an unresolved polytomy with other eastern European species (including *H. porrifolium*). Recent hybridization in *Hieracium* despite ample hybridization in the past, leading to thousands of allopolyploid apomictic species is rare, and hybrids are usually female sterile (Mráz et al., 2005, 2011). Besides, diploids are often endemic with very narrow distribution ranges and are sometimes known only from very few populations. Their distribution ranges and ecological requirements rarely overlap, and furthermore, the so-called mentor effect causes a breakdown of self-incompatibility under the influence of foreign pollen, which results in selfing and represents a strong barrier to introgression (Mráz, 2003; Mráz and Paule, 2006). For these reasons, if hybridization is responsible for the different placement of *H. vranceae* with different rDNA markers, it most probably did not occur recently. On the other hand, it is also difficult to explain the pattern by incomplete lineage sorting, because it should not show different relationships of the same individual with different, not closely related species with high support.

*Hispidella hispanica* is the only annual species of the Hieraciinae, endemic to central and western parts of the Iberian Peninsula. It is a monotypic genus that is, according to all molecular markers applied so far, sister to *Pilosella*. However, it showed a strongly divergent cloned 5S-NTS sequence that grouped near the base of *Hieracium*. This also is not a methodological artifact, because an indel position that differs between the clones was visible on the direct sequence, and the aberrant clone is very divergent from all other sequences of the Hieraciinae. We assume this represents yet another case of ancient intergeneric hybridization, this time not revealed by a discrepancy between rDNA regions or other molecular markers, but by a mixture of 5S-NTS variants at the intra-individual level. Only a 58 years old herbarium specimen was available for this species, and several attempts by us and a Spanish collaborator to collect *Hispidella* in the field failed, therefore the species can currently not be investigated in more detail. In *Potamogeton*, where ITS sequences are by far better homogenized than 5S-NTS sequences, the latter retained parental copies of hybrids even if the former have lost indications of hybrid origin or nearly so (Kaplan et al., 2018).

In the Mediterranean-Macaronesian genus *Andryala*, *A. integrifolia*, *A. glandulosa*, and *A. pinnatifida* belong to a ‘major radiation group’ with relatively recent speciation and largely unresolved species relationships (Ferreira et al., 2015). Within this group, three samples of *Andryala integrifolia* formed a well-supported monophyletic branch with ITS, but one phased allele of accession AZ 4 occurred in an unresolved position. In contrast, ETS and 5S-NTS grouped all alleles of two Algerian accessions (AZ 3/1 and AZ 4) whereas an Andalusian sample (JC 26/1) occurred outside this clade. With the low-copy nuclear marker *sqs*, the latter sample showed two rather divergent alleles, and generally, *A. integrifolia* was the most polyphyletic species of *Andryala* according to this marker (Ferreira et al., 2015). It is also known to hybridize with other species (Maire, 1937; García Adá, 1992), however, no indication for a recent hybrid origin of any of the accessions was found. Hybridization of the individuals of *A. integrifolia* studied here with *A. glandulosa* and *A. pinnatifida* can be excluded, because *A. glandulosa* is endemic to Madeira and *A. pinnatifida* to the Canary Islands. *Andryala integrifolia* is the most widespread species of the genus (Ferreira et al., 2015), which implies a larger population size. Here, rDNA patterns can be best interpreted as a consequence of recent divergence with incomplete lineage sorting.

In *Pilosella*, species relationships within clades a, c and d were unresolved, and their sequences were nearly identical within clades. In clade d, phased alleles of *P. echioides* and *P. onegensis* were more different than direct sequences of further species in the same clade with ITS and ETS, but this was not the case with 5S-NTS, where a fully homogenized sequence was found in *P. echioides* and two nearly identical phased alleles in *P. onegensis*. 5S-NTS showed a well-supported relationship of *P. echioides* with *P. cymosa*, but none of its divergent ITS or ETS alleles were grouping with that species. We assume that speciation in this clade was also recent and that incomplete lineage sorting is responsible for the differential behavior of the alleles. In contrast to *Hieracium*, recent hybridization in *Pilosella*, even across ploidy levels, is basically unlimited, and hybrids are usually fertile (Krahulcová et al., 2000; Fehrer et al., 2007b). Nevertheless, the morphologies of species in clade d are very divergent and no indications for introgression were observed in the material studied.

### Organization of 45S and 5S rDNA

Previous cytogenetic studies of the Hieraciinae focused on *Pilosella*, where an aposporous-specific meiotic avoidance locus and satellite markers were studied (Okada et al., 2011; Kotani et al., 2014; Belyayev et al., 2018) and on *Hieracium*, where satellite markers and rDNA loci of a few species were investigated (Ilnicki et al., 2010; Belyayev et al., 2018; Mráz et al., 2019; Chrtek et al., 2020; Zagorski et al., 2020). Additional species and populations that cover most of the phylogenetic lineages in both genera were added, and a species of genus *Andryala* was karyotyped here for the first time. *Andryala agardhii*, all *Pilosella* and the majority of *Hieracium* samples showed four loci of 45S rDNA and two loci of 5S rDNA per diploid genome, and their chromosomal organization – 45S rDNA in terminal positions and 5S rDNA in interstitial positions, the latter located on the same chromosome with one of the 45S rDNA loci – was the same. This indicates that this pattern represents the ancestral condition in the Hieraciinae. Furthermore, the same number and position of rDNA loci in diploids was inferred as the ancestral state across plants, except that the Hieraciinae have 18 chromosomes and the general karyotype of plants was inferred to have 16 chromosomes (Garcia et al., 2017).

In three species of *Hieracium* (*H. prenanthoides*, *H. lucidum*, and *H. sparsum*), six loci of 45S rDNA were found. The first two species belong to the western European clade, but *H. sparsum* belongs to the eastern European lineage. *H. prenanthoides* and *H. lucidum* form together a well-supported lineage of western European taxa in the 5S-NTS tree (Figure 3, A) and may therefore have acquired the additional 45S rDNA loci prior to their species divergence. In contrast, *H. sparsum* has apparently acquired the additional loci independently. Whether the possession of six loci is species-specific or not cannot be decided, however, without a broader geographic sampling of these species. The distribution areas of diploids (southwestern Alps in *H. prenanthoides*, Sicily in *H. lucidum* and the Balkans in *H. sparsum*) are relatively small so that these species may be actually uniform concerning the number of 45S rDNA loci. In *H. stelligerum*, another western European species, seven loci of 45S rDNA were observed. The ancestral state reconstruction based on the ITS/ETS tree implies a transition from 6 to 7 loci in the western European clade, but another possibility is a direct transition from 4 to 7 loci based on the position of *H. stelligerum* in the 5S-NTS tree (Figure 3, B). In this case, there is no indication whether the possession of seven loci is a species-specific pattern or not, because only a single sample was analyzed. The number of major polymorphisms in samples/species with four loci ranged from 0 (in *H. petrovae*, *H. pojoritense* PM2012, and *A. agardhii* JC 2011/31/1) to 17 (in *A. agardhii* A.agaJF and *P. onegensis* H1704); from 5 (in *H. sparsum* spa1611/5) to 31 (in *H. lucidum* Hluc_1-1-2) in samples with six loci (but a second sample of *H. lucidum* from the same population had only three polymorphisms); *H. stelligerum* Hstel_3-2-1 with seven loci showed 15 polymorphisms, and *H. umbellatum* UMB 8/9/3 with only three loci (see below) had six polymorphisms like another sample of the same species (H1617) that showed the usual four loci. The high variation in intra-individual sequence polymorphisms across samples, largely without any phylogenetic pattern, do not suggest consistent differences in the degree of homogenization of ITS or ETS sequences in relation to 45S rDNA locus number. Generally, interlocus concerted evolution seems to have operated fairly well in most samples, which may have been facilitated by the subtelomeric positions of the 45S rDNA loci (Wendel, 2000; Lan and Albert, 2011).

Within *H. sparsum* and also within *H. umbellatum*, the most widespread diploid *Hieracium* species (Bräutigam, 1992), intraspecific variation was observed. In *H. umbellatum*, one 45S rDNA locus was lost in accession UMB 8/9/3 from Slovakia, but not in accession H1617 from the Czech Republic. Their ITS and ETS sequences (representing the 45S rDNA) are identical. In *H. sparsum*, an additional locus of 5S rDNA was found in one accession from the Rila Mts whereas a second accession of that population and two accessions from the Pirin Mts showed the usual two loci of 5S rDNA. The additional locus of accession PM2099 (Figure 4E) occurred in an interstitial position on a chromosome not bearing also a 45S rDNA locus. 5S-NTS sequences from the variable population are not available (the plants perished), but two accessions of *H. sparsum* grouped together, albeit with low support. Intraspecific and even intra-population variation in the number of rDNA loci indicates that locus acquisition and loss can happen very quickly and also that it is not usable as a phylogenetic marker in the Hieraciinae. Also in many other plant groups, variation in the number and distribution pattern of rDNA is commonly observed among closely related species (Lan and Albert, 2011; Garcia et al., 2017; and references therein) and is therefore not informative concerning species relationships. Many studies in plants and animals have shown variation in rDNA locus number (e.g., Raskina et al., 2008; Gouja et al., 2015; Kolano et al., 2015; and references therein), suggesting that rDNA sites are highly dynamic components of the genome (Britton-Davidian et al., 2012).

Interestingly, three hemizygous loci were detected: seven loci and three loci of 45S rDNA in *H. stelligerum* and *H. umbellatum*, and three loci of 5S rDNA in *H. sparsum*. Hemizygous rDNA loci were also observed in other plant groups, for example in diploid and polyploid grasses (Rocha et al., 2018; Chiavegatto et al., 2019) and diploid orchids (Lan and Albert, 2011). Generally, a potential reason for the observation of hemizygous loci is hybridization (Myburg et al., 2003). However, none of the three *Hieracium* species (nor individuals) show any indication of recent or ancient hybridization, neither in their morphology nor with any molecular markers. Therefore, the occurrence of hemizygous loci, which were also frequently observed in other satellite DNAs in *Hieracium* (Belyayev et al., 2018; Zagorski et al., 2020), may have another reason. The genome size of *Hieracium* is approximately twice as high as that in *Pilosella* and *Andryala* (Suda et al., 2007; Chrtek et al., 2009; Zahradníček et al., 2018). This is suggestive of a whole genome duplication (WGD) that may have occurred in the ancestral lineage of *Hieracium*. WGD is widespread in the evolutionary history of the Asteraceae: In addition to a previously suggested paleopolyploidization event at the origin of the core-Asteraceae (Chapman et al., 2008, 2012), phylotranscriptomic analyses have uncovered at least four, possibly seven other events distributed at different levels in the Asteraceae phylogeny (Huang et al., 2016). A detailed genomic investigation of genus *Hieracium* is needed to understand if such an event has actually occurred in this genus as well and, if so, how it has affected its genome organization and the evolution of molecular markers.

## Conclusion

Molecular evolution of multi-copy sequences such as rDNA arrays poses specific challenges for phylogenetic inference. Appropriate treatment of intra-individual variation and the investigation of multiple markers can provide interesting insights in complex species relationships as well as in the evolution of the markers themselves. Contrary to most other plants, ITS and ETS sequences (45S rDNA locus) are more polymorphic than 5S-NTS sequences (5S rDNA locus) in Hieraciinae even though, generally, concerted evolution homogenized all rDNA arrays fairly well. Several strong discrepancies between ITS/ETS and 5S-NTS phylogenetic trees reveal previously unidentified cases of reticulation, and homogenization of the different arrays sometimes occurs in opposite directions. Comparison with the chromosomal organization of the loci corresponding to the markers shows that their location in the genome is far more dynamic than the sequences they contain, implying that chromosomal patterns are not suitable to infer species relationships, at least not in *Hieracium*.

## Data Availability Statement

The datasets presented in this study can be found in online repositories. The names of the repository/repositories and accession number(s) can be found below: https://www.ncbi.nlm.nih.gov/genbank/, MW315935–MW315953, MW325251–MW325296, MW328890–MW329033, MW587333–MW587351, and MW591759–MW591773; https://www.ebi.ac.uk/ena, ERS5458545–ERS5458563.

## Author Contributions

JF conceived of the study. JF and YB analyzed the data. YB developed the software tool. LP and RS did cytogenetic analyses. JJ did molecular labwork. JC and PM collected and determined the material. JF and YB wrote the manuscript. All the authors contributed to the drafts and gave final approval for publication.

## Conflict of Interest

The authors declare that the research was conducted in the absence of any commercial or financial relationships that could be construed as a potential conflict of interest.

## Footnotes

1 https://www.github.com/fephyfofum/quartetsampling

## Supplementary Material

Supplementary Figure 1 Phylogenetic analysis of the Hieraciinae based on the combined ITS and ETS regions. The Bayesian consensus tree is shown with posterior probabilities (pp) above branches and boostrap support (bs) from MP (regular) and ML (italics) analyses below branches. Values are only shown if pp was > 0.94 or bs > 70%. Below the support values, Quartet Concordance/Quartet Differential/Quartet Informativeness scores for 1000 replicates of the full alignment are displayed (in blue). Phased alleles are indicated behind accession labels as 0.0, 0.1, 1.0, 1.1., 0, 1, and s (single). These labels correspond to those in the ITS tree (Figure 1); swapped alleles for ETS are marked by asterisks (^∗^). d, direct sequence; a1/a2, two alleles of *Hispidella* (minor and major sequence inferred from direct sequencing); c, cloned sequence. W, E, western and eastern European clades of *Hieracium*. a-d, main lineages of *Pilosella*. Accession labels correspond to Table 1.

Supplementary Figure 2 Ancestral character state reconstruction on the maximum likelihood tree based on the combined ITS and ETS sequences using stochastic mapping of 45S rDNA loci (including taxa with unknown locus numbers). Locus numbers (see Table 1) were assigned to sequences (alleles) of the same individual. For *H. transylvanicum*, all further individuals were assigned four loci, because this species does not show intraspecific variation (Ilnicki et al., 2010). For *A. agardhii*, *P. lactucella*, *H. porrifolium*, and *H. vranceae*, for which only cytogenetic data from other individuals were available, locus numbers were assigned to the species. Pies at nodes represent the marginal ancestral states (empirical Bayesian posterior probabilities). Phased alleles are indicated behind accession labels as 0.0, 0.1, 1.0, 1.1., 0, 1, and s (single). Labels correspond to those in the ITS tree (Figure 1); swapped alleles for ETS are marked by asterisks (^∗^). For the tree with branch lengths and support values, see Supplementary Figure 1. d, direct sequence; a1/a2, two alleles of *Hispidella* (minor and major sequence inferred from direct sequencing); c, cloned sequence. W, E, western and eastern European clades of *Hieracium*.

Supplementary Table 1 Details of sample origins and voucher information.

Supplementary Table 2 Evolutionary transition model selection in a likelihood framework for stochastic character mapping using the corrected Akaike information criterion. ER, equal rates model; SYM, symmetrical model; and ARD, all-rates-different model. lnL, log-likelihood of the model; AICc, corrected Akaike information criterion; dAICc, difference between the AICc of the model and the best model; wAICc, AICc weight of the model. Differences in AICc between competing models were considered negligible if they were <3, very strong if >10, and moderately strong between 4 and 7 (Burnham and Anderson, 2002). The AICc weights indicate the probability that the model is the best among the whole set of candidate models.
