# Supplementary material for: Molecular Evolution and Organization of Ribosomal DNA in the Hawkweed Tribe Hieraciinae (Cichorieae, Asteraceae)
Source: Front Plant Sci. 2021 Mar 12;12:647375. doi: 10.3389/fpls.2021.647375 (PMC7994888; doi:10.3389/fpls.2021.647375)
Supplement: Supplementary Figure 1 — Phylogenetic analysis of the Hieraciinae based on the combined ITS and ETS regions. The Bayesian consensus tree is shown with posterior probabilities (pp) above branches and boostrap support (bs) from MP (regular) and ML (italics) analyses below branches. Values are only shown if pp was > 0.94 or bs > 70%. Below the support values, Quartet Concordance/Quartet Differential/Quartet Informativeness scores for 1000 replicates of the full alignment are displayed (in blue). Phased alleles are indicated behind accession labels as 0.0, 0.1, 1.0, 1.1., 0, 1, and s (single). These labels correspond to those in the ITS tree (Figure 1); swapped alleles for ETS are marked by asterisks (∗). d, direct sequence; a1/a2, two alleles of Hispidella (minor and major sequence inferred from direct sequencing); c, cloned sequence. W, E, western and eastern European clades of Hieracium. a-d, main lineages of Pilosella. Accession labels correspond to Table 1. [file Image_1.pdf]

Supplementary Figure 1 |
Phylogenetic analysis of the Hieraciinae based on the combined ITS and ETS regions.

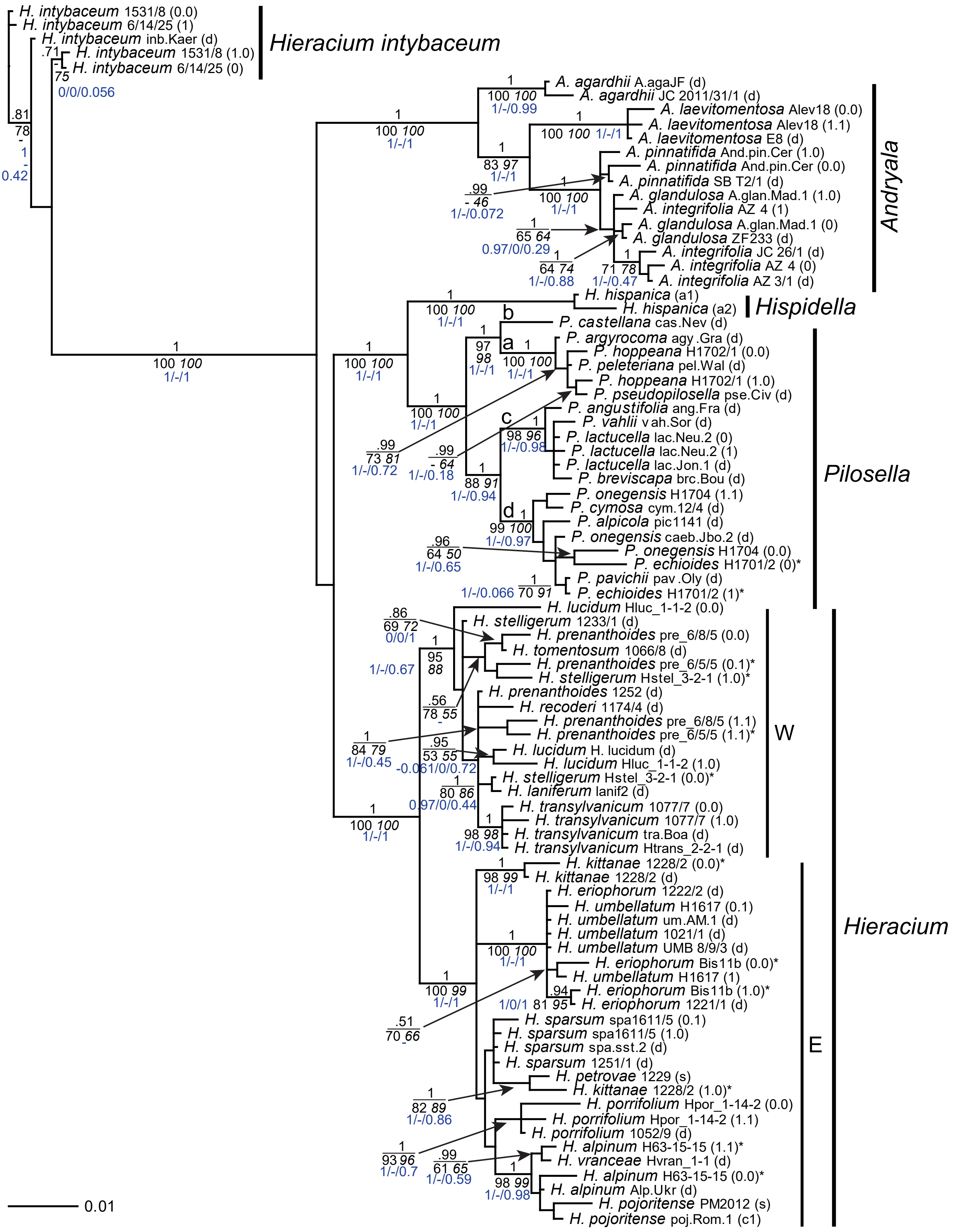

The Bayesian consensus tree is shown with posterior probabilities (pp) above branches and boostrap support (bs) from MP (regular) and ML (italics) analyses below branches. Values are only shown if pp was >0.94 or bs >70%. Below the support values, Quartet Concordance / Quartet Differential / Quartet Informativeness scores for 1000 replicates of the full alignment are displayed (in blue). Phased alleles are indicated behind accession labels as 0.0, 0.1, 1.0, 1.1., 0, 1 and s (single). These labels correspond to those in the ITS tree (Figure 1); swapped alleles for ETS are marked by asterisks (*). d – direct sequence, a1/a2 – two alleles of *Hispidella* (minor and major sequence inferred from direct sequencing); c – cloned sequence. W, E – western and eastern European clades of *Hieracium*. a-d: main lineages of *Pilosella*. Accession labels correspond to Table 1.
