# Supplementary material for: Molecular Evolution and Organization of Ribosomal DNA in the Hawkweed Tribe Hieraciinae (Cichorieae, Asteraceae)
Source: Front Plant Sci. 2021 Mar 12;12:647375. doi: 10.3389/fpls.2021.647375 (PMC7994888; doi:10.3389/fpls.2021.647375)
Supplement: Supplementary Figure 2 — Ancestral character state reconstruction on the maximum likelihood tree based on the combined ITS and ETS sequences using stochastic mapping of 45S rDNA loci (including taxa with unknown locus numbers). Locus numbers (see Table 1) were assigned to sequences (alleles) of the same individual. For H. transylvanicum, all further individuals were assigned four loci, because this species does not show intraspecific variation (Ilnicki et al., 2010). For A. agardhii, P. lactucella, H. porrifolium, and H. vranceae, for which only cytogenetic data from other individuals were available, locus numbers were assigned to the species. Pies at nodes represent the marginal ancestral states (empirical Bayesian posterior probabilities). Phased alleles are indicated behind accession labels as 0.0, 0.1, 1.0, 1.1., 0, 1, and s (single). Labels correspond to those in the ITS tree (Figure 1); swapped alleles for ETS are marked by asterisks (∗). For the tree with branch lengths and support values, see Supplementary Figure 1. d, direct sequence; a1/a2, two alleles of Hispidella (minor and major sequence inferred from direct sequencing); c, cloned sequence. W, E, western and eastern European clades of Hieracium. [file Image_2.pdf]

Supplementary Figure 2 | Ancestral character state reconstruction on the maximum likelihood tree based on the combined ITS and ETS sequences using stochastic mapping of 45S rDNA loci (including taxa with unknown locus numbers).

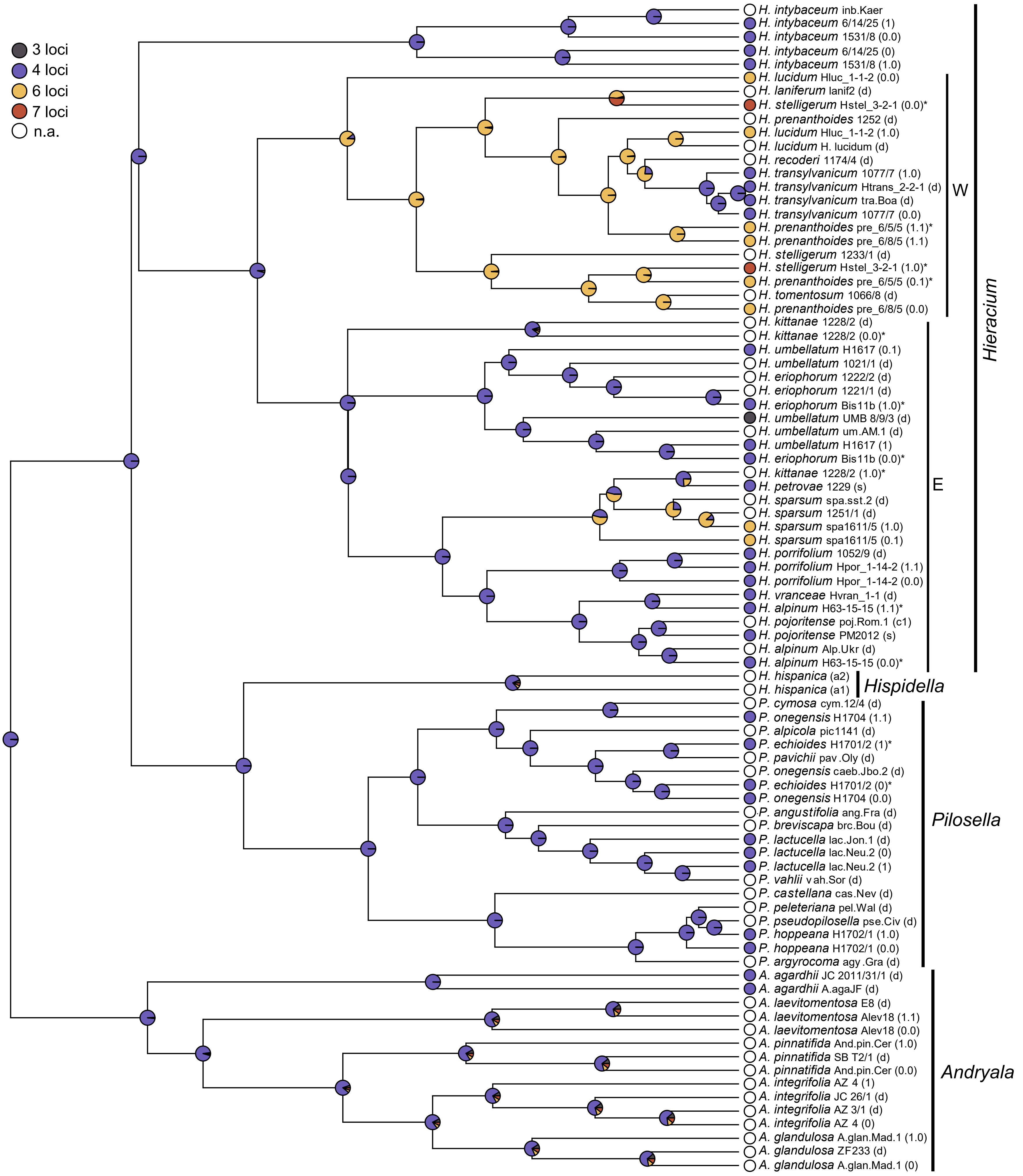

Locus numbers (see Table 1) were assigned to sequences (alleles) of the same individual. For *H. transylvanicum*, all further individuals were assigned 4 loci, because this species does not show intraspecific variation (Ilnicki et al., 2010). For *A. agardhii*, *P. lactucella*, *H. porrifolium*, and *H. vranceae*, for which only cytogenetic data from other individuals were available, locus numbers were assigned to the species. Pies at nodes represent the marginal ancestral states (empirical Bayesian posterior probabilities). Phased alleles are indicated behind accession labels as 0.0, 0.1, 1.0, 1.1., 0, 1 and s (single). Labels correspond to those in the ITS tree (Figure 1); swapped alleles for ETS are marked by asterisks (*). For the tree with branch lengths and support values, see Supplementary Figure 1. d – direct sequence, a1/a2 – two alleles of *Hispidella* (minor and major sequence inferred from direct sequencing); c – cloned sequence. W, E – western and eastern European clades of *Hieracium*.
