# Supplementary material for: Molecular Evolution and Organization of Ribosomal DNA in the Hawkweed Tribe Hieraciinae (Cichorieae, Asteraceae)
Source: Front Plant Sci. 2021 Mar 12;12:647375. doi: 10.3389/fpls.2021.647375 (PMC7994888; doi:10.3389/fpls.2021.647375)
Supplement: Supplementary Table 1 — Details of sample origins and voucher information. [file Table_1.pdf]

Supplementary Table 1 | Details of sample origins and voucher information.

Species	Identifier	Origin and voucher[1]
***Hieracium** alpinum*	alp.Ukr	Ukraine: Chornohora Mts., Ukrainian Carpathians, Polonina Breskulska ridge, saddle between Mt. Hoverla and Mt. Breskul, 1800 m, 48°09'09.8"N, 24°30'14.6"E, 23 July 2003, leg. P. Mráz & J. Chrtek, 0038/2003 (PRA)
	H63-15-15, H63-30-7	Ukraine: Zakarpattya, Mt. Blyznytsya, 1416 m, 48°25'N, 24°23'E, 28 July 2014, leg. J. Chrtek (PRA)
H. eriophorum	1221/1	France: dépt. Landes, Labenne: plage de Labenne Océan Sud, 10 m, 1°27'20"W, 43°36'17"N, 27 September 2006, leg. E. Forey, 1221/2006 (PRA)
	1222/2	France: dépt. Landes, Seignosse-le-Penon, plage de Estagnols Seignosse, 10 m, 1°25'51"W, 43°41'40"N, 27 September 2006, leg. E. Forey, 1222/2006 (PRA)
	Bis11b	France: dépt. Landes, Biscarrosse-Plage, 44°27'14"N, 1°15'09"W, August 2010, leg. D.J. Frey (MHNF)
H. intybaceum	inb.Kaer	Austria: Kärnten, Turracher Höhe, S. Jagalski 4 (M)
	1531/8	Austria: Tirol, Arlbergpass, 1970 m, 47°07'37.5190"N, 10°12'05.6430"E, 15 August 2015, leg. P. Mráz (PRA)
	6/14/25	France: Savoie, Col du Petit Saint-Bernard, 2060 m, 45°39'28.8"N 006°51'50.4"E, 28 August 2015, leg. P. Mráz (PRC)
H. kittanae	1228/2	Bulgaria: Central Rhodope Mts., Trigrad gorge, limestone rocks near the natural entrance to Dyavolskoto garlo cave, 750–800 m, 41°36'54"N, 24°22'45"E, September 2005, leg. P. Ignatova, 1228/2005 (PRA)
H. laniferum	lanif2	Spain: prov. Tarragona, la Sénia, 480 m, 40°40'15"N, 0°13'50"E, 4 June 2011, leg. J. Chrtek (PRA)
H. lucidum	H. lucidum	Italy: Sicily, distr. Palermo, Sferracavallo, limestone rocks between the village and Capo Gallo, 40 m, 13º17'56"E, 38º12'56"N, 11 April 2007, leg. J. Chrtek et al., 0054/2007 (PRA)
	Hluc_1-1-2	Italy: Sicily, Palermo, Mt. Gallo, 13°17'56"E, 38°12'56"N, 13 November 2014, leg. E. Di Gristina
H. petrovae	1229	Bulgaria: Central Rhodope Mts., Trigrad gorge, crevices of limestone rock (*locus classicus*), 750–800 m, 41°39'55"N, 24°21'50"E, 15 October 2005, leg. V. Vladimirov, 1229/2005 (PRA)
H. plumulosum	1218/2	Montenegro: Canyon of the Mrtvica river, 35 km SW of Kolasin, halfway through the canyon, near the bridge, 1000 m, 19°48'59"E, 42°28'40"N, August 2006, leg. J. Zahradníček, det. Z. Szeląg, 1218/2006 (PRA)
H. pojoritense	PM2012	Romania: Suceava, Pojorita, on the limestone scree of an abandoned quarry NE of the village, by the road E58, 690 m, 47°32'01.6"N, 25°29'43.9"E, 10 August 2014, leg. P. Mráz et al. (PRC)
	poi.Rom.1	Romania: Pojorita, Câmpulung Moldovenesc, 690 m, 47°32'01.6"N, 25°29'43.9"E, 27 July 2002, leg. P. Mráz & Z. Szeląg (PRC)
H. porrifolium	1052/9	Austria: Kärnten, Karawanks, Bad Eisenkappel, limestone rocks and pine forests (alliance *Erico-Pinion*) near the road to Bad Vellach, 4.5 km SSW of the town, 658 m, 14°34'20.5"E, 46°27'07.1"N, 26 July 2005, leg. J. Chrtek & P. Mráz, 1052/2005 (PRA)
	Hpor_1-14-1, -2	Slovenia: Upper Carniola, Podljubelj, 870 m, 46°25'31.8"N, 14°16'09.84"E, 3 September 2016, leg. P. Mráz (PRC)
	H1463	Slovenia: Julian Alps, Spodnja Trenta, Soča, 525 m, 46°21'24"N, 13°41'56"E, 4 October 2011, leg. B. Vreš (PRA)
H. prenanthoides	1252	France: dépt. Hautes Alpes, La Grave, below the village, ca 1500 m, 06°18'21"E, 45°02'37"N, June 2003, leg. P. Mráz, 1252/2003 (PRA)
	JC1513-3	France: Savoie, Modane, Villarodin, 45°12'27.0"N, 6°42'41.9"E, 24 August 2014, leg. J. Chrtek, M. Hartmann & M. Štefánek (PRA)
	pre_6/5/5, pre_6/5/2, pre_6/8/5, pre_6/4/5	Italy: Piemont, Claviere, 1570 m, 44°56'47"N, 6°46'09.1"E, 27 August 2015, leg. P. Mráz (PRC).
H. recoderi	1174/4	Spain: Catalunya, prov. Barcelona, Berga, monastery of Queralt, rocks ca 200 m below the parking place, 805 m, 1°49'24"E, 42°06'54"N, 24 July 2006, leg. J. Chrtek, 1174/2006 (PRA)

H. sparsum	1251/1	Bulgaria: Sofia, Vitoša Mts., NE slope of Mt. Vitoša, Bistriško Branište biosphere reserve, 2000 m, 23°17'56"E, 42°34'07"N, 23 June 2006, leg. F. Krahulec & A. Krahulcová, 1251/2006 (PRA)
	spa.sst.2	Bulgaria: Pirin Mts., Mt. Vihren, garden culture Z. Szeląg, 0034/2006 (PRA)
	spa1611/5, spa1611/6	Bulgaria: Pirin Mts., slopes of Mt. Vihren above the chalet, 41°45'26.5"N, 23°24'51.8"E, 16 August 2017, leg. J. Chrtek & K. Kabátová (PRA)
	PM2099, PM2102	Bulgaria: Rila Mts, Malyovitsa, 1800 m, 42°09'37.08"N, 23°22'54.84"E, 2017, leg. Z. Szeląg (Herb. Szeląg)
H. stelligerum	1233/1	France: dépt. Ardèche, Vallon Pont d'Arc, crevices of calcareous rocks along the road D 390, opposite of 'le Pont d'Arc', ca 3.5 km SE of the village, 500 m, 44°24'25"N, 04°24'10"E, October 2006, leg. P. Mráz, 1233/2006 (PRA)
	Hstel_3-2-1	France: dépt. Ardèche, Thueyts, Échelle de la Reine, 413 m, 44°40'12"N, 04°12'36"E, 4 May 2015, leg. P. Mráz (PRC)
H. tomentosum	1066/8	France: dépt. Alpes Maritimes, Roya Valley, Tende, along the old road to the Col de Tende, ca 0.5 km above the tunnel, 6 km NNW of the village, 1331 m, 44°08'19"N, 07°33'57"E, 28 August 2005, leg. J. Chrtek & P. Mráz, 1066/2005 (PRA)
H. transylvanicum	tra.Boa	Romania: Munţii Rodnei, border of the tourist path from the village of Borşa to Mt. Pietrosul Mare, spruce forest, 1300–1400 m, 47°39'N, 24°39'E, 5 July 2001, leg. P. Mráz (PRC)
	1077/7	Ukraine: Oblast Zakarpatska, Marmaroski Alps, Mt. Berlebashka, NW slope along the trail (red marked), E of the village of Dilove, 1200 m, 47°56'13"N, 24°21'31"E, 19 September 2005, leg. J. Zahradníček, 1077/2005 (PRA)
	Htrans_2-2-1	Romania: Hargitha, Băile Tuşnad, 730 m, 46°08'49.2"N, 25°51'03.6"E, 10 August 2014, leg. P. Mráz (PRC)
H. umbellatum	1021/1	Poland: Województwo pomorskie, Baltic coast, Jantar, 5 m, 54°20'N, 19°02'27"E, 27 June 2002, leg. Z. Szeląg, 1021/2002 (PRA)
	um.AM.1	Germany: Upper Lusatia, SE Schönau-Berzdorf, former brown coal mining area, slagheap, 51°03'22"N, 14°54'24"E, 20 August 2003, leg. S. Bräutigam (GLM 46889)
	H1617	Czech Republic: Praha, Troja, 50°7'10"N,14°25'15"E, 21 September 2017, leg. J. Chrtek (PRA)
	UMB 8/9/3	Slovakia: Košický region, Prakovce, 370 m, ,48°48'54"N, 20°54'43.2"E, 31 October 2015, leg. P. Mráz
H. vranceae	Hvran_1-1, PM2013	Romania: Vrancea, Cheile Tişiţei, on stones, sedimentary bedrock, 730 m, 45°55'18.0588"N, 026°33'59.22"E, 28 August 2013, leg. L. Filipaş & M. Bărbos (PRC)
***Pilosella** alpicola*	pic1141	Slovakia: Vysoké Tatry Mts., Mengusovská dolina valley, Satanov žľab, 1875 m, 49°10'01.6"N, 20°03'40.7"E, 12 August 2001, leg. P. Mráz & V. Mrázová (PRC)
P. angustifolia	ang.Fra	France: dépt. Upper Alps, S Col de Valbelle, 29 July1986, leg. M. Nydegger (GLM 155905)
P. argyrocoma	agy.Gra	Spain: prov. Granada, cultivated in Botanic Garden Munich, H. Merxmüller & W. Gleisner, culture H11
P. breviscapa	brc.Bou	France: Lac de Bouillouses, H. Merxmüller & B. Zollitsch 26985 (M)
P. castellana	cas.Nev	Spain: Sierra Nevada , above Monachil, 7 July 2000, leg. M. Nydegger (GLM 156412)
P. cymosa	cym.12/4	Czech Republic: Bohemia, Raná hill, 50°24'26.5"N, 13°46'19.0"E, 440 m, 12 May 2000, R. Šimek (PRA)
P. echioides	H1701/2	Czech Republic: Praha-Čimice, rocks above the Vltava river (Zámka), 50°08'38.3"N, 14°24'0.7"E , 18 July 2018, leg. J. Chrtek (PRA)
P. hoppeana	H1702/1	Austria: Kärnten, Hohe Tauern, Innerfragant, slopes of Mt. Bretterich, 1980 m, 46°57'29.5"N, 13°01'4.8"E , 28 July 1998, leg. J. Škorničková (PRA)
P. lactucella	lac.Jon.1	Germany: Jonsdorf, Oberlausitz, parking lot, 50°50'55"N, 14°42'12"E, June 1999, leg. S. Bräutigam (GLM 140619)
	lac.Neu.2	Germany: Erzgebirge, Neuwernsdorf, street at the dam, 50°41'58"N, 13°31'15"E, 17 June 2000, leg. S. Bräutigam (GLM 155665)
	Zebra	Czech Republic: Světlá nad Sázavou, cultivated in Botanical Garden of Charles University Prague, leg. J. Hadinec
P. onegensis	caeb.Jbo.2	Czech Republic: Giant Mts., Velká Úpa, Janovy Boudy, 50°41'28"N, 15°47'42"E, 1 July 1999, leg. S. Bräutigam (GLM

		156925)
	H1704	Czech Republic: Giant Mts, Trutnov, Velká Úpa, Pěnkavčí vrch, 988 m, 50°41'49.8"N, 15°47'21.9"E, 22 July 2017, leg. J. Doležal (herb. J. Doležal)
P. pavichii	pav.Oly	Greece: Mt. Olympos, A. Strid & S.O. Hansen 9638 (M)
P. peleteriana	pel.Wal	Switzerland: Kanton Wallis, Brig, old road towards Ganter bridge, 24 June 1996, leg. M. Nydegger (GLM 155337)
P. pseudopilosella	pse.Civ	Spain: prov. Civdad Real, Malagon, W Las Povedillas, 20 May 1994, leg. M. Nydegger (GLM 153084)
P. vahlii	vah.Sor	Spain: prov. Soria, Bot. Garden Munich, H. Merxmüller & W. Lippert, culture H43
***Hispidella** hispanica*	His.his.2	Spain: Sierra de Guadarrama, leg. J. Pizarro & C. Navarro (PR, no CN 2460)
***Andryala** agardhii*	JC 2011/31/1	Spain: Andalusia, province Granada, Sierra Baza, Calar del Desabezedo, 37°19'49"N, 02°51'20"W, 1195 m, 31 May 2011, leg. J. Chrtek & Z. Dočkalová, 1924/11 (PRA)
	A.agaJF, PM2390	Spain: garden culture, origin unknown (PRA)
A. glandulosa	A.glan.Mad.1	Portugal: Madeira, Ponta do Pargo, at the lighthouse, ca. 312 m, 32°48'51"N, 17°15'47"W, 4 November 2003, leg. S. Bräutigam (GLM 148659)
	ZF 233	Portugal: Madeira, Seixal, ca 42 m, 32°48'N, 17°04'W, 24 July 2009, leg. Z. Ferreira, 233 (MA)
A. integrifolia	AZ 4	Algeria: Algiers, town district Le Caroubier, 2 m, 36°44'N, 03°07'E, 3 July 2011, leg. A. Zeddam (PRA)
	AZ 3/1	Algeria: Algiers, town district Kouba, 90 m, 36°43'N, 03°05'E, leg. A. Zeddam, 678/12 (PRA)
	JC 26/1	Spain: Andalusia, province Granada, Guadix, 1075 m, 37°15'25"N, 03°08'16"W, 31 May 2011, leg. J. Chrtek & Z. Dočkalová, 1928/11 (PRA)
A. laevitomentosa	E8, Alev18	Romania: Suceava County, Bistrița Mountains, Mt. Pietrosul Bogolin, 1740 m, 47°22'37.1"N, 25°32'26.3"E, leg. J. Chrtek & al., 971/11 (PRA)
A. pinnatifida	SB T2/1	Spain: Canary Islands, Tenerife, Puerto de la Cruz, 780 m, 28°22'31"N, 16°36'37"W, 3 May 2010, leg. S. Bräutigam & E. Bräutigam, 2045/10 (PRA)
	And.pin.Cer	Spain: Canary Islands, La Gomera, Las Hayas, 1000 m, 28°07'N, 17°17'W, 17 April 2000, leg. S. Bräutigam (GLM 158131)

[1] For some accessions in PRA or PRC, the voucher is a plant of the same population.
