# Supplementary material for: Molecular Evolution and Organization of Ribosomal DNA in the Hawkweed Tribe Hieraciinae (Cichorieae, Asteraceae)
Source: Front Plant Sci. 2021 Mar 12;12:647375. doi: 10.3389/fpls.2021.647375 (PMC7994888; doi:10.3389/fpls.2021.647375)
Supplement: Supplementary Table 2 — Evolutionary transition model selection in a likelihood framework for stochastic character mapping using the corrected Akaike information criterion. ER, equal rates model; SYM, symmetrical model; and ARD, all-rates-different model. lnL, log-likelihood of the model; AICc, corrected Akaike information criterion; dAICc, difference between the AICc of the model and the best model; wAICc, AICc weight of the model. Differences in AICc between competing models were considered negligible if they were <3, very strong if >10, and moderately strong between 4 and 7 (Burnham and Anderson, 2002). The AICc weights indicate the probability that the model is the best among the whole set of candidate models. [file Table_2.pdf]

Supplementary Table 2 | Evolutionary transition model selection in a likelihood framework for stochastic character mapping using the corrected Akaike information criterion.

**Model**	**lnL**	**AICc**	**dAICc**	**wAICc**
**ER**	-27.8290	57.7605	0.0000	0.8940e
**SYM**	-23.7771	62.0248	4.2642	0.1060
**ARD**	-22.0641	79.2711	21.5105	0.0001

ER – equal rates model; SYM – symmetrical model; and ARD – all-rates-different model. lnL – log-likelihood of the model; AICc – corrected Akaike information criterion; dAICc – difference between the AICc of the model and the best model; wAICc – AICc weight of the model. Differences in AICc between competing models were considered negligible if they were < 3, very strong if > 10, and moderately strong between 4 and 7 (Burnham and Anderson, 2002). The AICc weights indicate the probability that the model is the best among the whole set of candidate models, selecting here the ER model.

Burnham, K. P., and Anderson, D. R. (2002). Model selection and multi-model inference: a practical information-theoretic approach. Springer, New York.
